# Supplementary material for: A robust method for on-chip production and manipulation of lipid vesicles by inverted emulsion
Source: Cell Rep Methods. 2026 Mar 10;6(3):101326. doi: 10.1016/j.crmeth.2026.101326 (PMC13030974; doi:10.1016/j.crmeth.2026.101326)
Supplement: Document S1. Figures S1−S4 [file mmc1.pdf]

Cell Reports Methods, Volume *6*

Supplemental information

A robust method for on-chip production and manipulation of lipid vesicles by inverted emulsion

Naresh Yandrapalli, David T. Gonzales, Weihua Leng, Cynthia Alsayyah, Nurzhan Abdukarimov, Robert Ernst, and T.-Y. Dora Tang

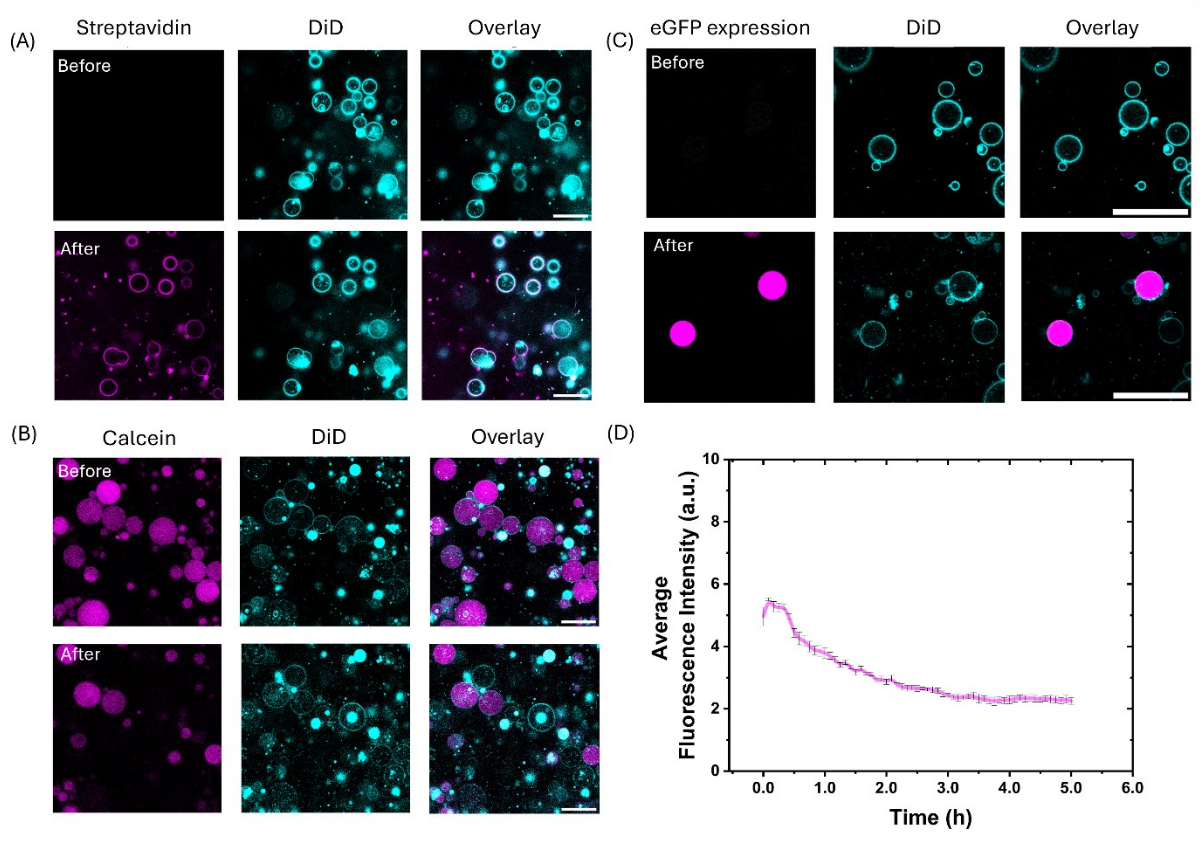

Figure S1: Diffusion-assisted manipulation of synthetic cells. (A) Confocal microscopy images of Streptavidin binding to vesicles with PE-biotin, before and after 30 min of incubation with 50 nM labelled Streptavidin protein. Scale bar – 50 µm. (B) Confocal snapshots of calcein containing vesicles before and after 3 h of incubation with 1µg/mL alpha-hemolysin membrane pore protein. Scale bar – 50 µm. (C) Confocal snapshots of b-CFES containing vesicles before and after 6 h of incubation with 1µM acyl-homoserine lactone. Scale bar – 50 µm. (D) Average luminal fluorescence intensity observed for vesicles with b-CFES that are uninduced. The timelapse plot suggests no increase in fluorescence intensity up to 5 h. Data is shown as mean ± SD. Related to Figure 3.

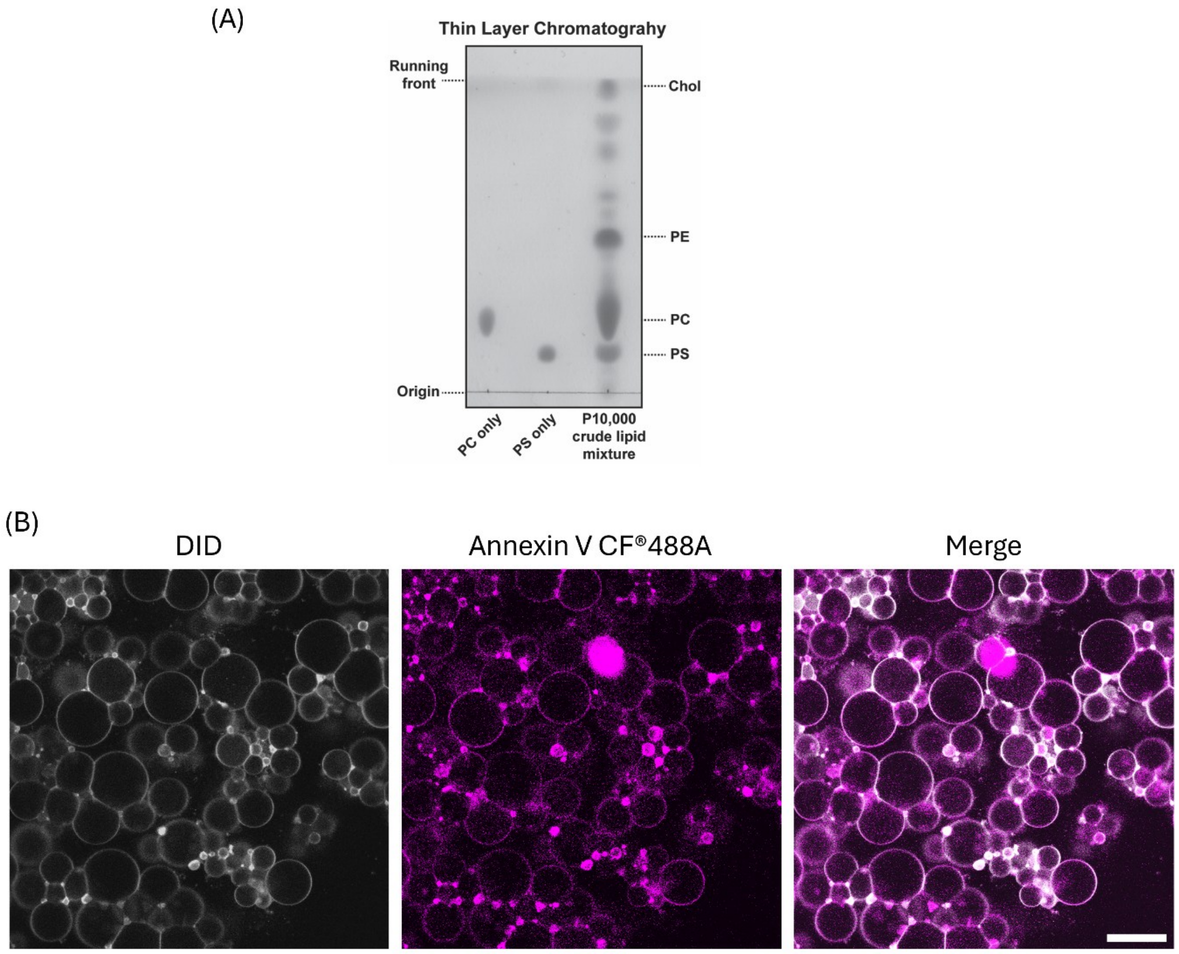

Figure S2: Synthetic cells production using mammalian lipid extracts. (A) Validation of crude lipid mixtures derived from HEK293T cells. Crude lipid extracts from mitochondria-enriched microsomes (P10.000) were subjected to TLC together with POPC (1 µL, 25 mg/mL) and POPS (1 µL, 25 mg/mL) and POPE (1 µL, 25 mg/mL) standards. Lipids were separated using chloroform:methanol:H_2O (70:25:2) as the mobile phase and stained with iodine. (B) Confocal image of bound Annexin V to PS containing synthetic cells with a lipid composition comprising of membrane pellet lipids extracted from mammalian HEK293T cells. Scale bar – 20 µm. Related to Figure 3

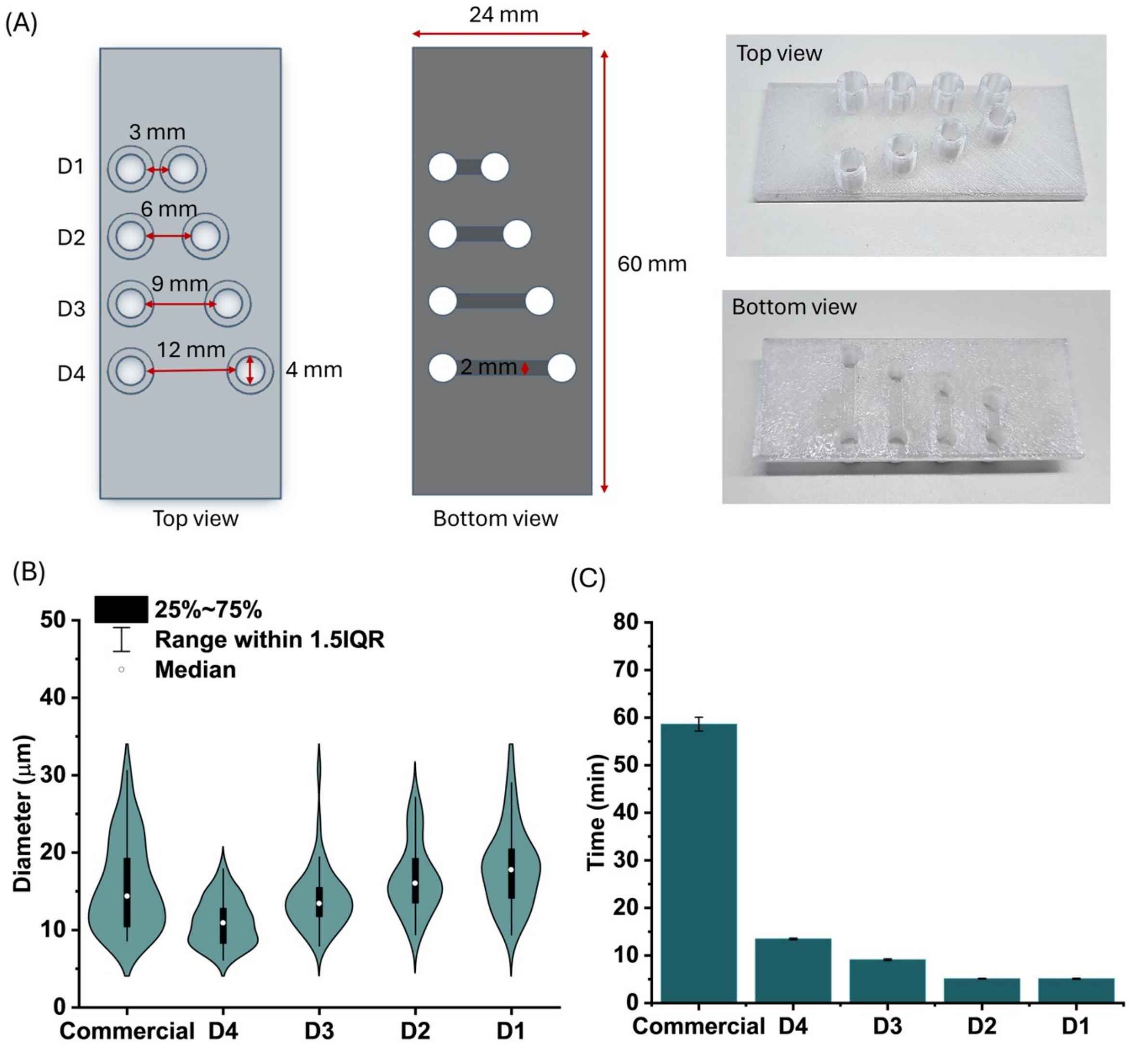

Figure S3: 3D printed chip design. (A) Schematics of the 3D printable chip design with varying channel lengths, D1 being the shortest and D4 the longest (left). Snapshots of 3D printed chips with top view and bottom view (left) after assembly with glass coverslip. (B) Comparative size analysis of size distribution of vesicles produced from commercial ibide® chips and 3D printed chip with four different distances are presented as violine plots obtained from n≥35. With a mean diameter around within 10-20 µm, has coefficient of variation, from left to right, 44%, 33%, 22%, 23% and 27%. (C) Diffusion time can be modulated depending on the length of the channel. The shorter the channel the faster the diffusion. Related Figure 1 & 2.

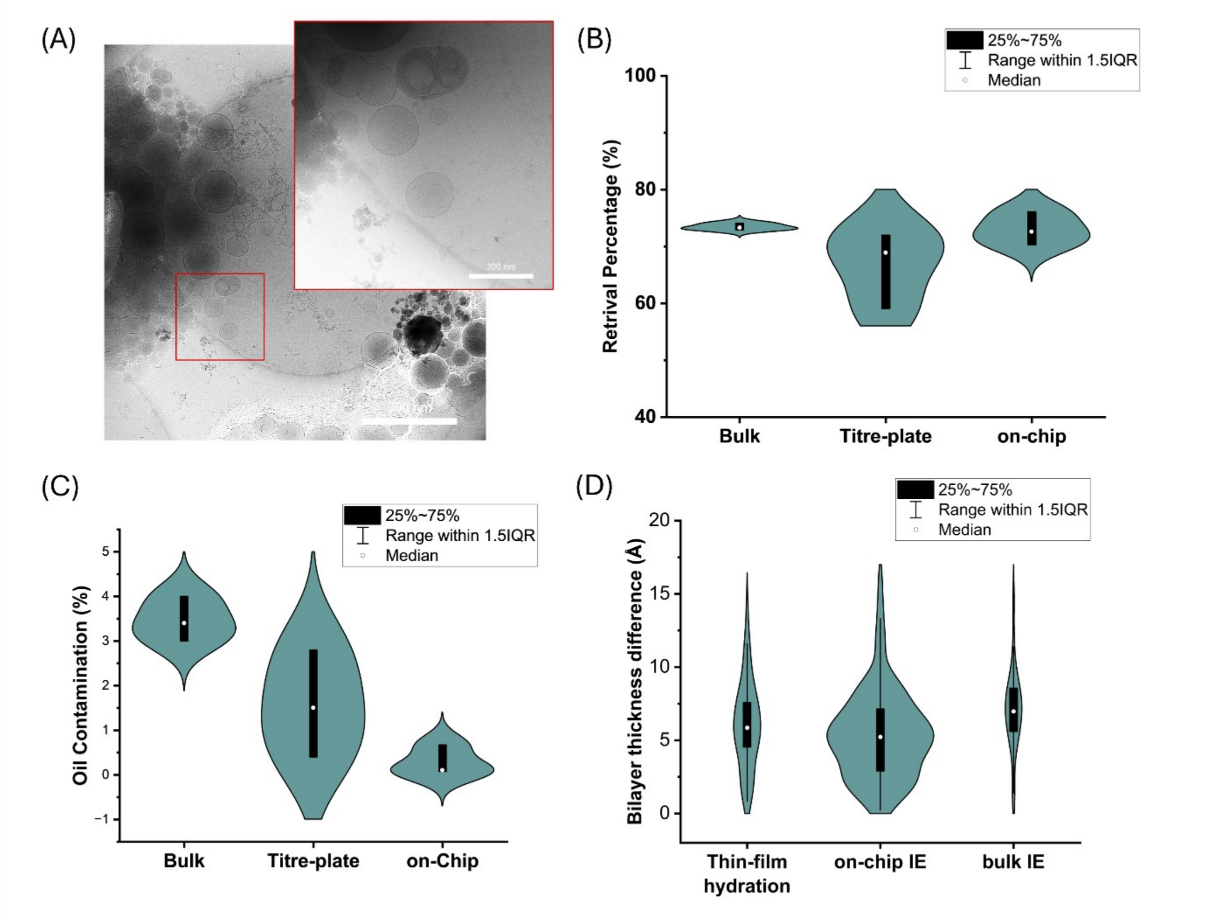

Figure S4: Systematic evaluation of synthetic cells after retrieval. (A) Wide field of view (11500x) electron micrograph of lipid vesicles, inset showing the close-up (48000x) of lipid vesicles. Scale bar corresponds to 300 nm. (B) Comparative flow cytometry analysis of number of vesicles retrieved from Eppendorf tubes vs Microtitre 96-well plate vs on-chip methods. The average retrieval percentage corresponds to 73.4±0.6%, 66±6%, and 73±2.9% for all the methods, respectively. In each of these cases vesicles were removed through an oil layer. Our results show that vesicles are lost during retrieval through oil regardless of the vessel they are contained. **(C).** DiD-based percentage oil contamination of vesicles retrieved using three different methods was evaluated using Flow cytometry. The average contamination from retrieval via an oil layer from an Eppendorf®-based method (bulk) or microtiter 96-well plate (titre plate) showed 8.8±0.98% and 4.1±2.5% contamination respectively. Retrieval from the second well of the chip (on chip) without an oil layer showed 0.97 ± 1.2% contamination. The violine plots are obtained using three replicates for all three methodologies. (D) Distribution of the bilayer thickness difference amongst vesicles produced using different methods. The on-chip IE and thin-film hydration methods produce membranes with a lower median asymmetry (~5-6 Å) compared to the bulk IE method (~7 Å) (n≥50). Related to Figure 4.
