# Supplementary material for: A robust method for on-chip production and manipulation of lipid vesicles by inverted emulsion
Source: Cell Rep Methods. 2026 Mar 10;6(3):101326. doi: 10.1016/j.crmeth.2026.101326 (PMC13030974; doi:10.1016/j.crmeth.2026.101326)
Supplement: Document S2. Article plus supplemental information [file mmc10.pdf]

Cell Reports
Methods

Article

A robust method for on-chip production and manipulation of lipid vesicles by inverted emulsion

Graphical abstract

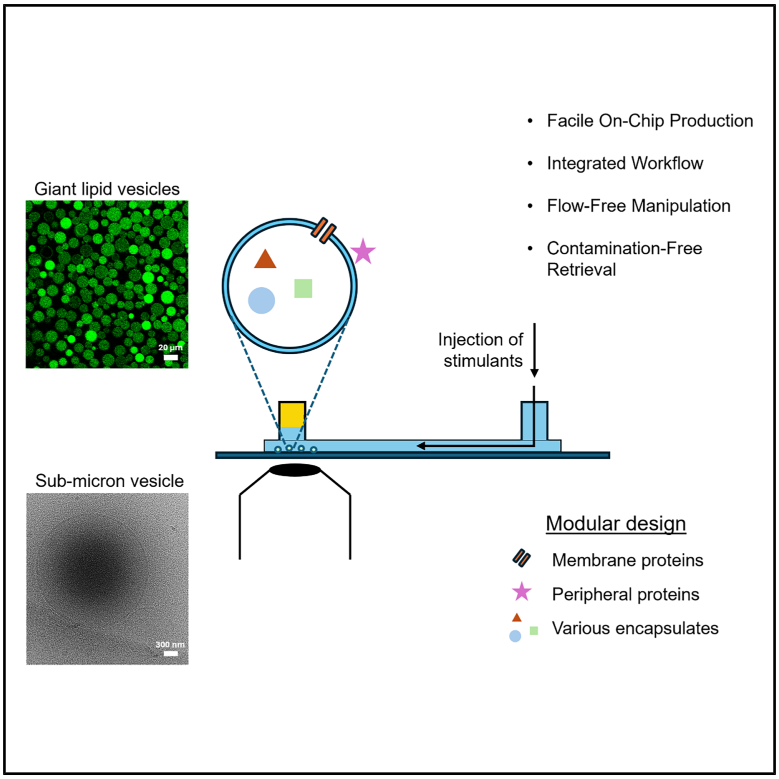

Authors

Naresh Yandrapalli, David T. Gonzales, Weihua Leng, Cynthia Alsayyah, Nurzhan Abdukarimov, Robert Ernst, T.-Y. Dora Tang

Correspondence

naresh.yandrapalli@uni-saarland.de (N.Y.),
dora.tang@uni-saarland.de (T.-Y.D.T.)

In brief

Yandrapalli et al. present an on-chip inverted emulsion method that integrates vesicle production, manipulation, and analysis in one platform without the removal of vesicles through the oil layer. This approach reduces vesicle contamination and loss while enabling diverse biochemical assays, thereby lowering the barriers for synthetic cell and membrane research.

Highlights

- A single-chip method for lipid vesicle preparation, manipulation, and imaging
- Enables retrieval of vesicles while avoiding the oil phase
- Avoids specialized microfluidics and associated shear forces
- Low-cost, accessible workflow suitable for broad synthetic biology applications

Yandrapalli et al., 2026, Cell Reports Methods *6*, 101326
March 23, 2026 © 2026 The Author(s). Published by Elsevier Inc.
https://doi.org/10.1016/j.crmeth.2026.101326

CellPress

Cell Reports Methods

CellPress
OPEN ACCESS

Article

A robust method for on-chip production and manipulation of lipid vesicles by inverted emulsion

Naresh Yandrapalli,[1,2,*] David T. Gonzales,[1] Weihua Leng,[1] Cynthia Alsayyah,[3] Nurzhan Abdukarimov,[4] Robert Ernst,[3] and T.-Y. Dora Tang[1,2,5,*]
[1]Max Planck Institute of Molecular Cell Biology and Genetics, Pfotenhauerstrasse 108, 01307 Dresden, Germany
[2]Department of Synthetic Biology, Gebaude B2.2, University of Saarland, 66123 Saarbrücken, Germany
[3]Center for Molecular Signaling, Medical Biochemistry & Molecular Biology Department, University of Saarland, 66421 Homburg, Germany
[4]INM-Leibniz Institute for New Materials, 66123 Saarbrucken, Germany
[5]Lead contact
*Correspondence: naresh.yandrapalli@uni-saarland.de (N.Y.), dora.tang@uni-saarland.de (T.-Y.D.T.)
https://doi.org/10.1016/j.crmeth.2026.101326

MOTIVATION Conventional inverted emulsion methods for the preparation of lipid vesicles are hampered by multiple intervention steps from preparation to characterization and use in assays. We address this problem by developing an inexpensive, easy-to-fabricate device with connected wells on a glass slide that allows for lipid vesicle production, manipulation, and imaging on one chip.

SUMMARY

Lipid vesicles are important as minimal model systems for cellular compartmentalization. They drive major advances in deciphering biological mechanisms by molecular reconstitution; provide rational solutions for primitive compartmentalization in origin-of-life studies; form the basis of synthetic cells and drug delivery vehicles. The emulsion method is a well-established route for producing bilayer lipid vesicles. However, the application of this method in microfluidics requires complex and specialized machinery. The bulk method suffers from the need to physically manipulate the vesicles through oil layers for characterization that can damage the vesicles. Given this, we present a facile and robust method for on-chip production and manipulation of lipid vesicles by the emulsion method. We prepared a simple device that allows preparation, imaging, and collection of activated lipid vesicles. This technique combines minimal processing steps with maximum flexibility in lipid vesicle production and manipulation with direct imaging, thus fast-tracking production lines across disciplines.

INTRODUCTION

Synthetic cells are important technologies for addressing the grand challenge of building a minimal living cell from scratch and can provide a step toward the development of new materials; provide models for understanding biological systems; and have been demonstrated to support directed evolution.[1,2] The basis of synthetic cells are a membrane bound compartment that can be based on biological molecules such as lipids[3,4] or synthetic molecules and macromoleculs such as polymers[5,6] or a combination of both.[7,8] These can then be activated at the membrane[9] or be used to encapsulate biological machinery for gene expression[10,11] or cell division.[12–14] Further, generation of nested vesicle structures provide simplified models for intracellular organization.[15,16] This has consequently, fueled the development of methodologies for reproducible and controllable liposome or polymerosome formation in the past decades. Emulsion-based methods for the preparation of liposomes are popular due to their ability to encapsulate diverse molecules along with the ability to use different lipid compositions and buffers in a facile manner.[17,18] Among various emulsion-based techniques such as microfluidics-based double emulsion method[19] and continuous droplet interface crossing encapsulation (cDICE),[20] the inverted emulsion method is considered to be a robust methodology with minimal infrastructural and technical expertise requirements.[18,21,22] In brief, microfluidics-based double emulsion methods involves the use of precision flow in micrometer-sized channels to yield homogeneously sized vesicles. cDICE uses micro-meter-sized glass capillaries and a rotating chamber with lipid-containing oil, and water layers. Water-oil emulsions are injected into the rotating chamber to yield vesicles. The inverted emulsion method includes simple preparation of sonicated

© 2026 The Author(s). Published by Elsevier Inc.
This is an open access article under the CC BY license (http://creativecommons.org/licenses/by/4.0/).

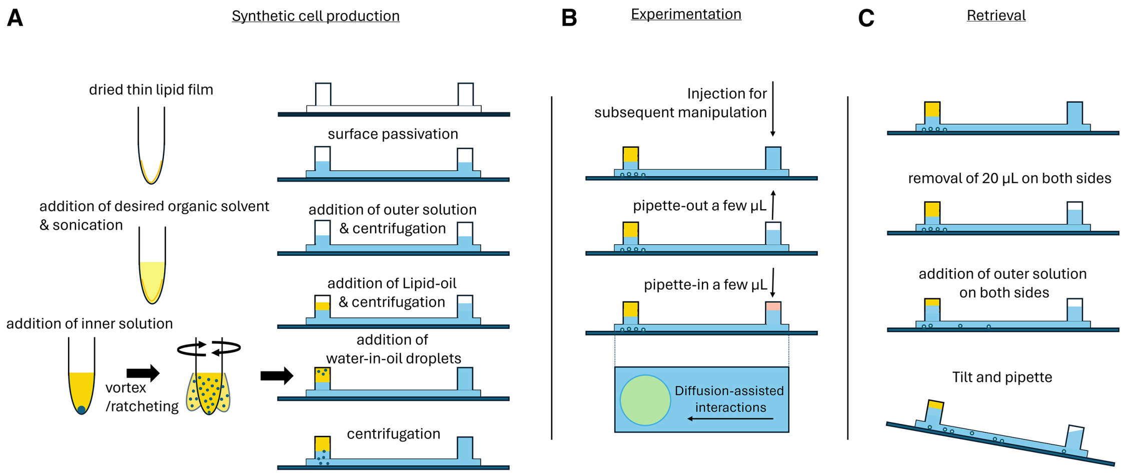

Figure 1. Single-device production, visualization, and retrieval of lipid vesicles via the inverted emulsion method

(A) Detailed schematic of vesicle production, from lipid-containing mineral oil dispersion to surface coating of the chip and layering of outer solution with the lipid-oil followed by formation of water-in-oil droplets and centrifuge to form lipid vesicles.

(B) Schematic showing simple introduction of biomolecules into the chamber for easy manipulation of produced lipid vesicles within the chip.

(C) Schematic detailing the retrieval process of lipid vesicles from the chip for subsequent analysis.

lipid-mineral oil solutions, and centrifugation of preformed water-in-oil (W/O) droplets. It can be undertaken by "bulk" methodologies using standard microtiter well plates[18] or within Eppendorf tubes[23] with typical volumes of 500 mL (for the latter) and short (30 min) preparation times.

This method has been used to establish symmetric as well as asymmetric bilayers using bacterial lipids[24]; incorporate enzymatic catalysis[25,26]; study the effect of cytoskeletal networks on membrane shape[27]; initiate communication between synthetic and natural cells[28] and between two different synthetic cell populations[29,30]; and for drug delivery applications.[22,31] Despite its widespread use there remain challenges in using this standard method, most significantly in the use of the lipid vesicles for imaging or for further assays. In this case, the vesicles need to be removed from the lower water layer through the oil layer to be transferred to another vessel for the assay. This can disrupt the lipid vesicles and is a major bottleneck of the "bulk" method. Microfluidics provides an alternative route to the preparation of lipid vesicles that utilizes small volumes of liquids and materials. However, executing microfluidics require specific infrastructure and specialized devices from pressure/syringe pumps to fabrication in clean rooms.[32–34] This may not be ideal for standard biochemistry or biophysics lab where such facilities may not be available.

To provide a robust platform for lipid vesicle preparation which does not require any specialized equipment, we have lowered the requirements and simplified the production, examination, and manipulation of synthetic cells while addressing some of the major bottlenecks in implementing the inverted emulsion method. For this, we use a commercially available two-well chambered slide from Ibidi (Figures 1 and 2A). The device design allows for the application of inverted emulsion technique using a total volume of 160 μL. With a two-well design connected via a channel, this device allows for the preparation of lipid vesicles in one well of the channel that can be imaged directly without the requirement to remove the vesicles and load them into a separate sample holder (Figure 1A). The second well on the device provides a route to access the vesicles without interacting with the oil layer. Moreover, biomolecules placed in the adjacent well can interact with vesicle membranes through diffusion (Figure 1B). Finally, the vesicles can be extracted, when required, without passing through the oil phase via the use of the second well (Figure 1C). With a glass bottom, this chambered slide allows for direct imaging and manipulation of vesicles without the relocation of vesicles after preparation and avoids any flow-induced interactions that arise from the use of microfluidic traps. This approach avoids the disruptive shear forces inherent in conventional flow-based microfluidic systems[35] and the vesicle removal step in bulk method for assaying. To demonstrate the utility of the method, we validated the stability of the prepared vesicles and performed diffusion-assisted protein-lipid binding and activation of incorporated cell-free gene expression. Together, this one stop device provides a cost-effective alternative for the preparation, manipulation, and optical characterization of lipid vesicles that can be used for a broad range of applications.[36]

RESULTS

Vesicle preparation via the inverted emulsion method in chambered slides

To make lipid vesicles, we prepared a device from readily available commercial laboratory consumables (Ibidi μ-Slide VI 0.5 Glass Bottom) (Figure 2A). The chambered slides have two wells connected via a ~15 mm long channel and have a total volume of 160 μL. We show that the inverted emulsion method can be implemented within this device. First, the surface of the glass slide is passivated with β-casein to prevent vesicle collapse upon wetting the glass surface. The chamber is rinsed to remove excess, unbound protein, and then filled with the outer solution; this is

CellPress
OPEN ACCESS

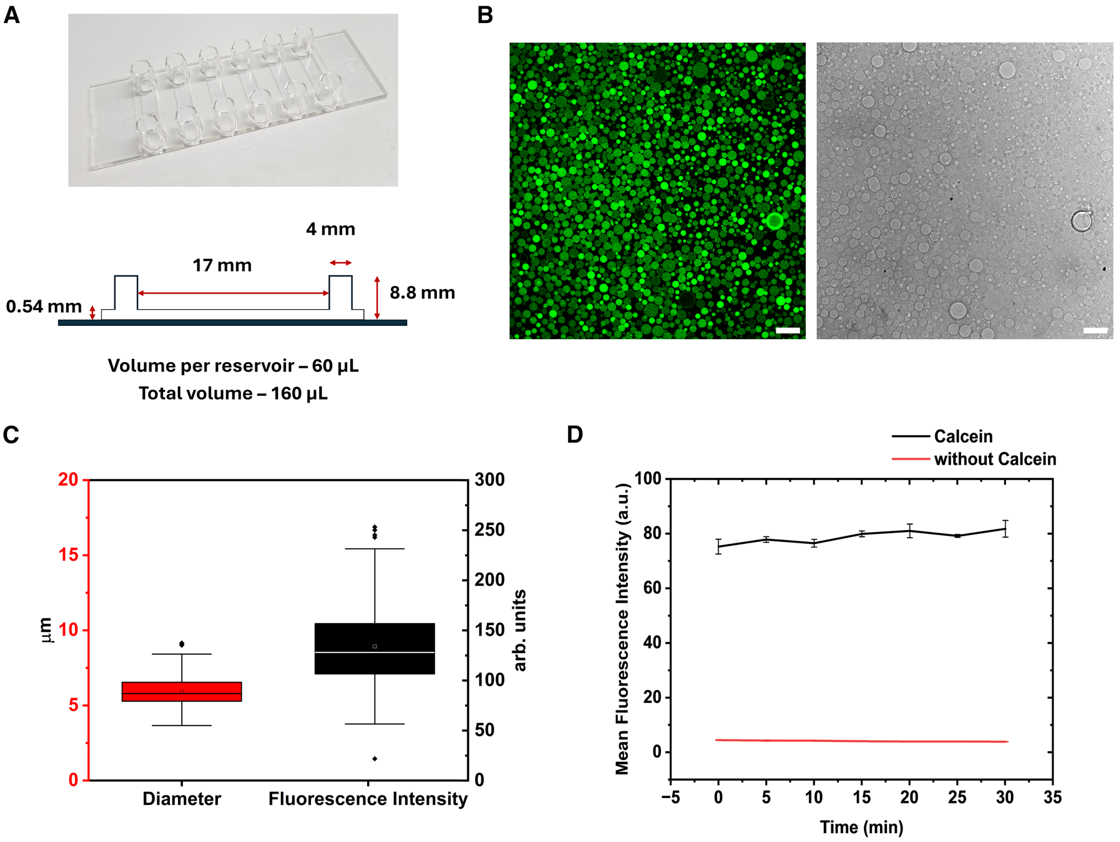

Figure 2. Lipid vesicle production on-chip

(A and B) (A) Schematic and image of channel device; (B) confocal cross-sectional image of synthetic cells (dioleoylphosphatidylcholine, DOPC 99.5%, 1,1′-dioctadecyl-3,3,3′,3′-tetramethylindodicarbocyanine perchlorate, DiD 0.5%) filled with calcein (10 μM) and bright-field image of the vesicles; scale bars, 20 μm.

(C) Boxplot depicting the size (5.89 ± 1.03 μm in diameter) and calcein encapsulation distribution (133 ± 43, 30% variance) within vesicle population ($n \geq 100$).

(D) Average luminal fluorescence intensity observed for vesicles with 10 μM encapsulated calcein (black line) and without calcein (red line). The time-lapse plot suggests no fluctuations in fluorescence intensity over a period of 30 min. Data points are an average of total fluorescence intensity across a field of view for three experiments. Data are represented as mean ± SD.

See also Video S1.

typically a buffer (with or without sugars). The oil phase containing the lipid (produced through sonicating of the desired concentration of lipid in mineral oil as shown in Figure 1A) is added to one well and incubated at room temperature for 30 min to allow the formation of a lipid-monolayer at the oil-water interface (refer to STAR Methods).

After the addition of the oil phase in one well, the same volume of outer solution was added to the second well to prevent fluid movement between the channels. During the 30-min incubation period, the W/O emulsions are prepared. For this, the inner aqueous solution (5 μL) of the vesicle is pipetted into an Eppendorf tube containing lipid-oil (200 μL) and physical agitation methods such as vortexing or ratcheting is exerted on the solution. The inner solution is broken down into smaller droplets that are stabilized by lipid. The size of these water-in-oil emulsions determines the size of the vesicles. To produce smaller vesicles (<1 μm), the W/O droplets can be further subjected to magnetic stirring (2,500 rpm, for 10 min, used for preparing vesicles for cryoEM imaging [see Figure 4]). 25 μL of the W/O dispersion is then added to well 1 containing the lipid-oil dispersion. The same volume of outer buffer is added to the other well (well 2) to maintain the pressure. Immediately, the slide is centrifuged (refer to Table 1) with swinging buckets to help transfer the lipid-stabilized W/O droplets through the preformed lipid monolayer at the interface of lipid-oil to form vesicles (Figure 2B). The slide can then be directly placed onto a microscope stage for imaging. Analysis of the vesicles (Figure 2C) showed a large number of vesicles with a mean diameter of 5.89 μm and a coefficient of variance, 17.48% that is typical of vesicles prepared by the emulsion method within microtiter plates.[18] Similarly, the encapsulation efficiency and vesicle stability over time is studied using a calcein-based encapsulation assay (Video S1). The calcein fluorescence distribution within the vesicle population has a variance of ~30% (Figure 2C) which is comparable to previous studies,[18] and the vesicles are stable with no sign of calcein leakage over 30 min (Figure 2D). With this standardized methodology, we systematically tested the utilization of different

Table 1. Conditions for the inverted emulsion method in this study

Inner solution	Outer solution	Centrifugation speed (×g) for 3 min
Cell-free extract	see Table 4	2,500
10 μM calcein +600 mOsm sucrose	600 mOsm glucose	1,500
600 mOsm sucrose	600 mOsm glucose	1,500

See also Figures 2 and 3.

lipid composition and integration with various biomolecules to confirm the viability of this approach for standard applications.

Protein immobilization to vesicles

The ability to control membrane composition and properties has been shown to be important for membrane biophysical studies.[37] The method described here is amenable to changing the lipid composition of the final vesicles at the preparation steps. By exploiting the second well, one can add biomolecules to interact with the vesicles without any need to extract the vesicles or remove the oil layer (Figure 3A). The flexibility in our methodology allowed the demonstration of membrane-protein interactions by using labeled streptavidin and Annexin V proteins. We tuned the lipid composition to contain 0.5% biotinylated dioleoylphosphatidylethanolamine (DOPE) lipids and 99.5% DOPC lipids. After the preparation of the lipid vesicles, 1 μL of streptavidin (15 μM) tagged with Alexa Fluor 488 was loaded into the second well to achieve a final concentration of 100 nM. Immediately after injection, the solution in well 2 was mixed by repeated pipetting to facilitate molecular diffusion. Given the design of the device, the pipetting action in well 2 does not disrupt or disturb the vesicles in well 1 as compared to conventional bulk methods. After 10 min, the vesicles in well 1 were imaged by fluorescence microscopy and an equatorial cross-section of the lipid vesicles was obtained (Figures 3A, 3B, and S1A; Video S2). The line profile shows co-localization of the fluorescence signal of lipid analog, DiD, and streptavidin. Within the observed sample, 100% of the vesicles showed protein binding suggesting homogenous interaction of biotinylated lipids among all the population. Similarly, to validate the potential of the methodology in using complex lipid mixtures and physiologically relevant biomolecules we used mammalian HEK293T cell lipid extracts to prepare lipid vesicles that contain PS lipids (Figure S2A). The vesicles are labeled with Annexin V CF 488A (100 nM) that bind PS lipids in the presence of calcium ions (4 mM) (Figures 3C, 3D, and S2B). Annexin V is a well-known biomarker in apoptosis and known to play an important role in mending lipid membrane in the presence of extracellular calcium.[38,39] Our results showed that our method is amenable to preparing lipid vesicles from mammalian cell extracts. Further, the use of calcium in vesicle formation resulted in clustered vesicles (Figures 3C and S2B) due to its ability to bridge phosphate groups across lipids.[40] However, line profile analysis shows clear binding between the labeled Annexin V protein and PS-containing lipid vesicles (Figure 3D). Together, the presented data demonstrate that this technique is effective in generating lipid vesicles from controlled lipid compositions and cell extracts. In addition, the vesicles can be activated by biomolecules that are introduced to the system using the second well without disruption to the prepared vesicles.

Membrane protein-based material transfer across membrane

Membrane protein reconstitution into lipid bilayers is an important method for deciphering the structural and functional properties of membrane proteins as well as to ascertain the nature of the lipid bilayer. As such, we demonstrate that the lipid vesicles prepared within the channel devices can be activated with a membrane protein, to release internalized cargo. In this case, we prepared lipid vesicles containing calcein (10 μM) with a composition of DOPC:Chol—60%: 20% along with 20% of DHPC for efficient incorporation of alpha-hemolysin. This lipid composition with unsaturated acyl chains and short chain detergent molecules are well suited for the incorporation of membrane spanning pore proteins such as alpha-hemolysin.[41] After the preparation of lipid vesicles, alpha-hemolysin was added to the solution from the second well. After approximately 20 min, direct imaging of the lipid vesicles within the chamber showed a decrease in fluorescence intensity within the lumen of the vesicles which is commensurate with calcein leakage from the vesicles (Figures 3E, 3F, and S1B; Video S3). The kinetics of calcein leakage followed an exponential decay, indicating a first-order release process (see STAR Methods). The model provided an excellent fit to the experimental data (adjusted R^2 = 0.986), confirming a first-order release process. From this fit, we determined a characteristic time constant (τ) for the leakage of 29.96 ± 0.82 min. Further control experiments, i.e., in the absence of alpha-hemolysin showed no calcein dye leakage from the vesicles over a 3 h period (Figure 3F). The exponential decay in fluorescence intensity within the lumen of the SCs is observed (n = 10), ∼20 min after the addition of the protein. However, unlike previous studies where the full decay in fluorescence intensity is completed within ∼30 min, we observed a longer decay timescale. This is due to the usage of lower protein concentration compared to other studies as well as the time required for alpha-hemolysin to diffuse through the channel. In this case, we wanted to simply demonstrate that leakage can be driven in the chip without removal of the vesicles. The onset and time course of the leakage could be tuned by changing the length of the channel (see Figure S3) or alpha-hemolysin concentration, respectively, where we assume that higher concentrations of protein pore would lead to a faster leakage.[32,42]

Chemical activation of vesicles

One of the major advantages of the inverted emulsion methodology is to be able to encapsulate complex sets of biomolecules. Here, we encapsulate the home-made bacterial cell-free expression system[29] supplemented with feed mix (see Table 2) and chemically induce its expression via an inducer molecule that is supplemented into the oil-free well (well 2). The inducer molecule can diffuse through the channel to the vesicles to induce expression of a reporter gene (Figures 3G, 3H, and S1C). To demonstrate this, we prepared W/O emulsions using 5 μL of inner solution (see Table 3) containing cell-free expression system (prepared from *E. coli* cells) and plasmid DNA (pT7-Lux R and

CellPress
OPEN ACCESS

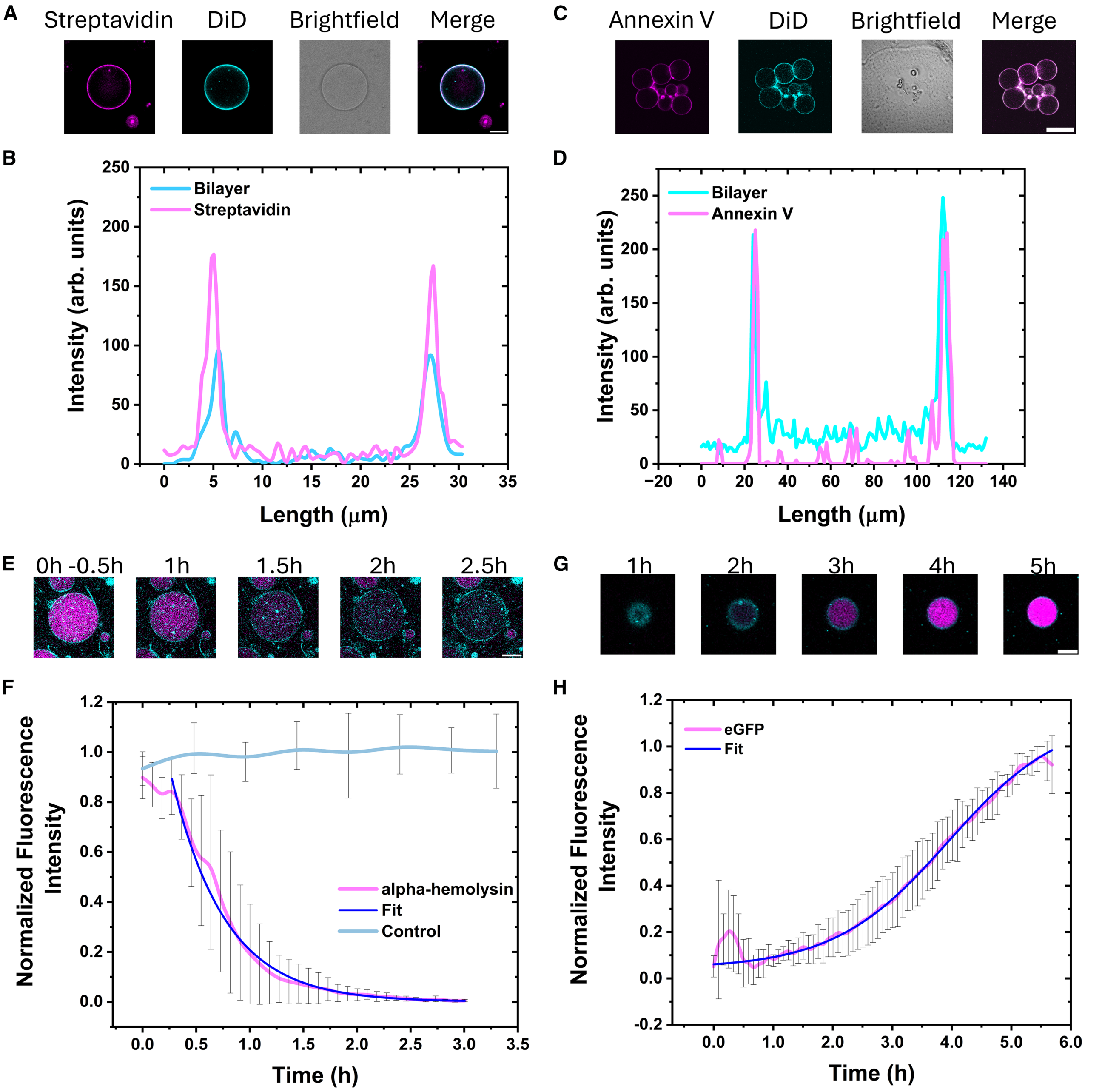

Figure 3. Diffusion-based manipulation of lipid vesicles

(A) Step-by-step schematic representation of ligand injection into the chambered slide for vesicle manipulation.

(B) Confocal cross-sectional snapshots of streptavidin protein binding to the lipid membrane (DOPC:Biotin PE:DiD—99%:0.5%:0.5%) of the vesicles, along with bright-field image showing distinct contract (suggesting, no leakage). Scale bars, 10 μm.

(C) Confocal cross-sectional snapshots of Annexin V protein binding to the PS-containing lipid membranes (mammalian lipid extract) in the presence of calcium ions. Scale bars, 10 μm.

(D) Line profile graph representing the overlap of distinct peaks from both the dyes, suggesting colocalization of Annexin V and the lipid membrane.

(E) Confocal cross-sectional snapshots of material (calcein) leakage from synthetic cells (DOPC: dihexanoylphosphatidylcholine, DHPC: cholesterol, Chol—60%:20%:20%) after the addition of alpha-hemolysin (1 μg/mL). Scale bars, 10 μm.

(F) Plot depicting the exponential decrease in fluorescence intensity over time upon membrane pore formation (n = 10). Data are represented as mean ± SD.

(G) Confocal cross-sectional snapshots of synthetic cell (DOPC:DID—99.5%:0.5%) with bacterial cell-free expression system expressing enhanced green fluorescent protein (EGFP), triggered by injection of acyl-homoserine lactone, final concentration of 100 nM. Scale bars, 10 μm.

(H) Plot depicting the gradual increase in fluorescence intensity within the lumen of the vesicle (n > 5). Data are represented as mean ± SD.

See also Videos S2, S3, S4, and S5.

Table 2. List of reagents used in solution A and solution B

Solution A		Solution B	
Component	Final (mM)	Component	Final (mM)
ATP	12.4	Amino acid mix	14.3
GTP	8.7	Magnesium glutamate	71
CTP	8.7	Potassium glutamate	930
UTP	8.7	PEP (pH 7)	238
Folinic acid	0.68	–	–
tRNA	0.176 mg/mL	–	–
NAD	2.7	–	–
CoA	1.8	–	–
Oxalic acid	27.2	–	–
Putrescine	6.8	–	–
Spermidine	10.1	–	–
HEPES (pH 7)	774.5	–	–

See also Figure 3.

Table 3. bCFES mixture (inner solution) for synthetic cell preparation

Component	μL
Solution A	1.8
Solution B	1.75
bCFES	5
MgGlu (200 mM)	0.156
pT7 LuxR	0.625
pLux EGFP	0.625
Water	1.25

See also Figure 3.

pLux-GFP and 200 μL of lipid-oil dispersion (DOPC:DID—99.5%:0.5%) in an Eppendorf tube. These W/O emulsions subsequently form lipids vesicles by centrifugation through the oil-water interface in well 1 in the chamber slide. We use a simple gene circuit, as previously described[29]: LuxR is constitutively expressed via the T7 promoter and binds to the chemical inducer, acyl-homoserine lactone (AHL). Together, this complex binds to the pLux promoter to activate eGFP gene expression. We took advantage of the ability of AHL to cross the lipid bilayer to trigger EGFP production within the vesicles by adding AHL to the outer solution via well 2. To achieve this, 10 μL of the outer solution (see Table 4) was replaced with 10 μL of 2 μM AHL solution to achieve a final concentration of 100 nM. As the device allows for direct imaging of the vesicles after preparation, we directly placed the device on a microscope stage. Fluorescence imaging of the lipid vesicles shows the onset of fluorescence intensity from GFP after 1 h with continual increase in fluorescence intensity up to 6 h (Figures 3H and S1C; Video S4) showing that we were able to induce gene expression within the device. Importantly, the vesicles remained intact with no signs of membrane destabilization during 6 h of expression. The resulting expression kinetics followed a characteristic sigmoidal curve (Figure 3H), featuring an initial lag phase of approximately 1.5 h, followed by a rapid increase in fluorescence. Fitting the data to a Boltzmann sigmoidal function (see STAR Methods) yielded a half-maximal expression time (t_{50}) of 3.94 ± 0.06 h, that provides an approximate timescale of the entire process from induction to protein production. This sigmoidal profile, with its distinct lag phase, is characteristic of complex, multistep biological processes like transcription and translation, confirming the successful operation of the gene circuit within the vesicles.[29] In the absence of AHL, there is no observable gene expression (Figure S1D; Video S5).

Retrieval and visualization of the synthetic cells

One of the advantages of this chamber device is the ability to prepare and image the lipid vesicles without any physical intervention. However, in some cases, it may be necessary to remove lipid vesicles for further characterization or assaying. In both microtitre well-plates and Eppendorf tube-based methodology, vesicle retrieval can be a delicate process due to the proximity of oil which can contaminate or disrupt the vesicles. This can be circumvented in our chambered slide by tilting the device toward well 2, allowing the vesicles to move by flow and removing vesicles from the device without oil contamination (Figure 1C). Simultaneous pipetting from the well will allow the retrieval of the vesicles from the chamber. Alternatively, 20 μL of oil is removed from the second well and the same volume is loaded into the first well. This will facilitate/force the movement of the vesicles toward the second well before the retrieval step. Given that the volume of the chamber is (~160 μL), the entire volume of synthetic cells can be removed while avoiding the oil. To show that the vesicles were intact after removal, we collected the vesicles and loaded them onto an EM grid for imaging by cryo-TEM. Cryo-TEM was used to determine the lamellarity of the vesicles.[43] We note that for these experiments, the vesicles were isolated, frozen, and stored for a week, retaining their integrity over this time frame. The micrographs produced show no visual evidence of residual oil and the bilayer contrast is qualitatively similar to that of vesicles produced using thin-film hydration method (Figures 4B, 4D, and S4A). Our results also showed intact vesicles with a differential distribution of unilamellar (88%) and multilamellar vesicles (12%) for our on-chip methodology (Figure 4C). A comparative analysis of the vesicles generated using on-chip inverted emulsion method with bulk, Eppendorf-based inverted emulsion method, and thin-film hydration showed a larger % of multilamellar vesicles produced in the bulk method (17%) compared to on-chip protocol (12%). The thin-film hydration method produced a low number of multilamellar vesicles (2%) (Figure 4C) as the vesicles were subjected to sonication (20 min) which is a standard method to increase the unilamellarity of the sample. Despite this, the results indicate that between the inverted emulsion methods the on-chip method provide a slight advantage over bulk method with regard to multilamellarity.

To confirm that vesicles removed through an oil later would damage the vesicles, we used flow cytometry to quantify the % of vesicles within a population which might have been affected by retrieval through an oil layer. Vesicles doped with 1% NBD dye prepared by the thin film hydration method were loaded into an Eppendorf tube, well plate and on-chip devices. While the retrieval of vesicles from Eppendorf tubes and well plates was via a mineral oil layer (doped with 5% DiD label), our on-chip methodology has a second connected well that provides an oil-free

Table 4. bCFES mixture (outer solution) for synthetic cell preparation

Components	mM
ATP	5
CTP	1.5
UTP	1.5
GTP	3
Amino acids mix	0.5
Spermidine	1.5
DTT	1.5
Folinic acid	0.02
K-glutamate	280
Mg-glutamate	20
HEPES	150
Glucose	200

See also Figure 3.

access to the vesicles. Once retrieved, the vesicles are analyzed for DiD contamination. Flow cytometry data showed some loss in the vesicles when extracted via oil layers (Figure S4B) and a higher contamination of vesicles was observed for Eppendorf-based method (8.8 ± 0.98%) and microtiter 96-well plates (4.1 ± 2.5%) compared to the on-chip methodology (0.97 ± 1.2%) (Figure S4C).

For a quantitative comparison, we analyzed the mean bilayer thickness of vesicles produced using the thin-film hydration method after sonication to vesicles produced using both on-chip and Eppendorf tube (bulk IE)-based inverted emulsion methods using CryoVia image analysis toolkit.[44] Our results (Figures 4E and S4D) indicate on-chip IE method produced vesicles with a median membrane thickness of approximately 28 Å. This value closely mirrors the median thickness of vesicles created via thin-film hydration (~30 Å). In contrast, vesicles formed using the bulk IE method were distinctly thicker, with a median thickness of approximately 33 Å. The high-density region of the on-chip IE plot, representing the most common vesicle structure, shows a strong overlap with the profile from thin-film hydration. This trend was also evident when analyzing the bilayer thickness difference, a measure of membrane thickness asymmetry (Figure S4D). The median asymmetry for on-chip IE vesicles (~5 Å) were again highly comparable to that of thin-film hydration vesicles (~6 Å). Both showed lower median asymmetry than the bulk IE method (~7 Å), indicating that the on-chip method produces a population of vesicles whose structural properties are most similar to those created by the classical thin-film hydration technique. Furthermore, we show that removal of the vesicles from the second well of the chip leads to vesicles that have a lower contamination of the oil compared to conventional methods. This provides a clear advantage for the on-chip method over the standard bulk methods.

DISCUSSION

This work presents a simplified approach to lipid vesicle production (see Video S6) and manipulation that has the potential to advance research in synthetic biology; biochemical reconstitution and biophysics by providing a simple and robust method for vesicle formation. In summary, the results show the preparation of stable lipid vesicles with a mean diameter of 5.89 μm and a low coefficient of variance (17.48%), which demonstrate that this method produces highly homogeneous vesicles with limited but high homogeneity in encapsulation efficiency (~70%) (Figure 2C). Over a period of 6 h, these vesicles demonstrated good stability (Video S5) that is important for their use cases in a wide range of applications including the study of protein-lipid interactions, membrane protein incorporation studies, and cell-free protein expression (Figure 3). We have shown binding of peripheral proteins such as Annexin V proteins to lipids membranes prepared from mammalian cell extracts. While alpha-hemolysin is an especially robust membrane protein for membrane activation, our results show that protein can be added into the device with minimal perturbation while incorporating into the vesicle to induce leakage. Together, this suggests that this device is an ideal platform for optimization of lipid composition and for detergent or detergent-free membrane protein reconstitution. In principle, lipids and proteins which are amenable to vesicular formation and *in vitro* manipulation, respectively, are compatible with this method. The broader significance of these results lies in their potential to accelerate progress in the fields of synthetic biology and membrane biophysics where vesicle formation and unperturbed manipulation are key for studies requiring high precision, such as those involving diffusion-based signaling or molecule transfer.

That said, subsequent extraction and analysis of the vesicles play an important role in understanding further characterization. We have shown that the extraction, flow cytometric analysis and cryo-TEM imaging of produced vesicles is feasible (Figure 4). To our surprise, approximately 12% of the vesicles showed a multilamellar membrane structure (Figure 4C)—potentially due to the presence of excess lipid or oil where extraction of the oil during centrifugation can lead to multilayered structure. Nevertheless, the produced vesicles have a membrane structure comparable to vesicles generated via a simple hydration technique.

The chambered slide offers ease of use, cost-effectiveness, and availability that makes this method accessible to a wide variety of laboratories. A brief analysis of the costs indicates that preparing these devices from commercially available channels will cost less than 4 Euros for a single use experiment. This is more costly than preparing vesicles by the inverted emulsions methods in an Eppendorf tube where one tube is approximately 50 cents. However, the latter does not offer the advantages provided by the channel device. It is possible that the cost of the experiment using the channel device can be reduced by 3D printing the channels (see Figure S3) (.stl file is attached as a supplemental file, Data S1). Barring the initial cost of the 3D printer (approximately 1,000 Euros) the cost of an experiment can be reduced to less than 30 cents.

Future extensions to these custom-built chips are readily accessible: (1) reduction of the channel length could allow for lower material volume and diffusion time (see Figure S3); (2) increase throughput and efficiency can be achieved by implementing the system in a 96-well plate with connected wells; (3) immobilization assays can be achieved by surface modifications

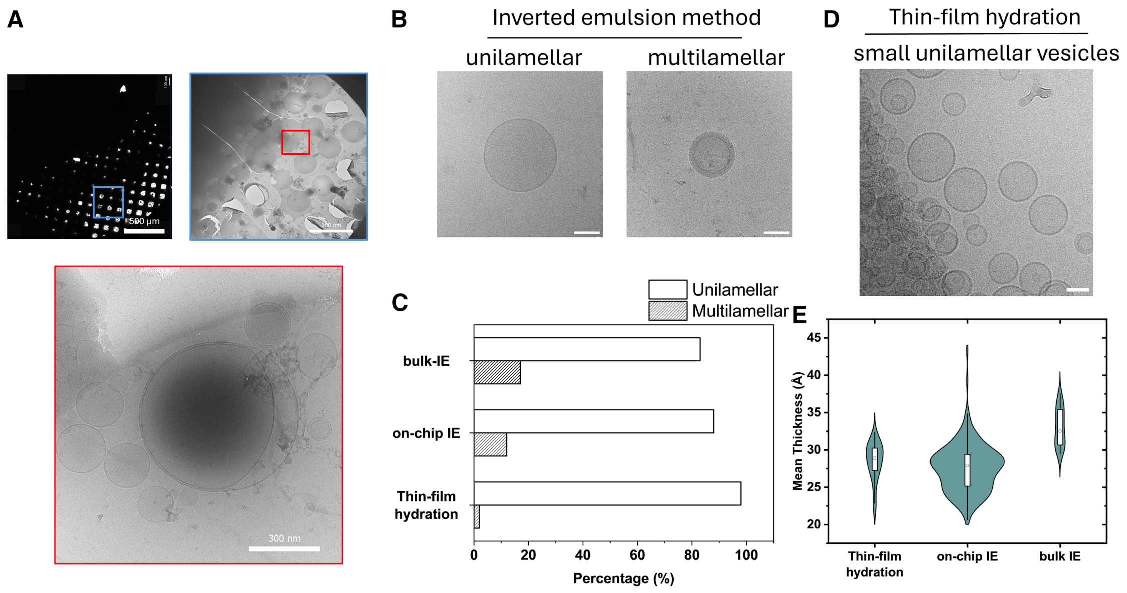

Figure 4. Lipid vesicle retrieval

(A) Cryo-TEM workflow showing whole grid image (top) with insert showing the multiple wells and vesicles (bottom).

(B) Cryo-TEM micrographs of synthetic cells, with unilamellar (left) and multilamellar (right) architectures. Scale bars, 50 nm.

(C) Bar chart showing number of multilamellar vesicles to unilamellar vesicles obtained in all three methods tested. Analysis was undertaken on $n \geq 100$ liposomes across 4 different experiments. Samples were loaded onto 2–3 grids of 50 holes.

(D) Cryo-TEM micrographs of small unilamellar vesicles produced using the thin-film hydration method with sonication. Scale bars, 50 nm.

(E) Violin plot comparing the mean bilayer thickness of vesicles produced using the thin-film hydration method, the on-chip inverted emulsion method, as well as the inverted emulsion method in Eppendorf tubes ($n \geq 50$).

of the glass slide prior to adhering to the chip; and (4) the chip design can still benefit from a more intuitive retrieval process, further enhancing its potential for studying complex biological phenomena in minimal models.

Limitations of the study

Despite the advantages, the study has some limitations. While the method provides a low-tech alternative to microfluidic devices, it may not yet achieve the same level of precision in terms of vesicle size control, for example. The reliance on centrifugation and passive diffusion may limit the control (movement in-presence of heat flux, for example) researchers have over vesicle behavior in comparison to pressure-driven microfluidic systems. In addition, despite our TEM images showing comparable membrane thicknesses to the hydration method there may still be small traces of oil in the membrane which could hinder their use in biological assays. However, the benefits of the methodology out perform its limitations and can be used for a wide variety of applications including biological assay. This method is especially effective for laboratories with no expertise in microfluidics and is interested in encapsulating biomolecules within lipid vesicles and further perturbing the system using the built-in diffusion channel.

RESOURCE AVAILABILITY

Lead contact

Requests for further information and resources should be directed to and will be fulfilled by the lead contact, T.-Y. Dora Tang (dora.tang@uni-saarland.de).

Materials availability

The custom-built chambered slide design in.stl format is included as a supplemental file. Plasmids have been deposited in AddGene (see method details).

Data and code availability

- Data has been deposited at Mendeley Data and is publicly available as of the date of publication at https://doi.org/10.17632/rx7bkm4mtb.1.
- This study does not report original code.
- Any additional information required to re-analyze the data reported in this paper is available from the lead contact upon request.

ACKNOWLEDGMENTS

We thank the MPI-CBG, Dresden; Universität des Saarlandes and the PharmaScienceHub; and the European Research Council (ERC consolidator grant, MINSYNCELL, grant agreement no. 101088834) for financial support. R.E. and T-Y.D.T. acknowledge funding from the SCALE Excellence Cluster of the Deutsche Forschungsgemeinschaft. The authors are grateful for the services and facilities of MPI-CBG and for the outstanding support provided, notably the Electron Microscopy Facility.

AUTHOR CONTRIBUTIONS

N.Y. and T.-Y.D.T. conceptualized the work; N.Y. designed and performed all the experiments; D.T.G. provided the plasmids; N.Y. and N.A. performed flow cytometry experiments; C.A. and R.E. provided the lipid extracts from mammalian cells; N.Y. and W.L performed and analyzed cryo-TEM measurements; and N.Y. and T.-Y.D.T. wrote the paper.

DECLARATION OF INTERESTS

The authors declare no competing interests.

CellPress
OPEN ACCESS

STAR★METHODS

Detailed methods are provided in the online version of this paper and include the following:

- KEY RESOURCES TABLE
- METHOD DETAILS
  - *E. coli* extract
  - *E. coli* cell-free extract preparation
  - Solution A
  - Solution B
  - The CFES reaction mix
  - Preparation of lipid extracts from mammalian cell culture
  - Isolation of whole cell lipids from HEK293T cells
  - Isolation of lipids from mitochondria-enriched, crude microsomes
  - Total lipid extraction
  - Thin-layer chromatography (TLC)
  - Inverted emulsion method
  - Thin film hydration with sonication
  - Confocal microscopy
  - Electron microscopy
  - 3D printable chip production
  - Analysis of vesicle loss and contamination due to extraction through the oil phase
- QUANTIFICATION AND STATISTICAL ANALYSIS

SUPPLEMENTAL INFORMATION

Supplemental information can be found online at https://doi.org/10.1016/j.crmeth.2026.101326.

Received: August 8, 2025
Revised: December 1, 2025
Accepted: January 21, 2026
Published: March 10, 2026

REFERENCES

1. Adamala, K.P., Dogterom, M., Elani, Y., Schwille, P., Takinoue, M., and Tang, T.Y.D. (2023). Present and future of synthetic cell development. Nat. Rev. Mol. Cell Biol. *25*, 162–167. https://doi.org/10.1038/s41580-023-00686-9.

2. Tang, T.-Y.D., Huang, X., Tang, T.-Y.D., and Huang, X. (2023). Artificial Cells: From Basic Assembly to Directed Functionality. Small Methods *7*, 2301446. https://doi.org/10.1002/SMTD.202301446.

3. Justice, I., Kiesel, P., Safronova, N., von Appen, A., and Saenz, J.P. (2024). A tuneable minimal cell membrane reveals that two lipid species suffice for life. Nat. Commun. *15*, 9679. https://doi.org/10.1038/s41467-024-53975-y.

4. De La Serna, J.B., Perez-Gil, J., Simonsen, A.C., and Bagatolli, L.A. (2004). Cholesterol Rules: DIRECT OBSERVATION OF THE COEXISTENCE OF TWO FLUID PHASES IN NATIVE PULMONARY SURFACTANT MEMBRANES AT PHYSIOLOGICAL TEMPERATURES. J. Biol. Chem. *279*, 40715–40722. https://doi.org/10.1074/JBC.M404648200.

5. Seo, H., and Lee, H. (2024). Programmable Enzymatic Reaction Network in Artificial Cell-Like Polymersomes. Adv. Sci. *11*, e2305760. https://doi.org/10.1002/advs.202305760.

6. Yandrapalli, N., and Antonietti, M. (2022). Dewetting-Assisted Interface Templating: Complex Emulsions to Multicavity Particles. Adv. Sci. *9*, 2203265. https://doi.org/10.1002/advs.202203265.

7. Go, Y.K., and Leal, C. (2021). Polymer-Lipid Hybrid Materials. Chem. Rev. *121*, 13996–14030. https://doi.org/10.1021/acs.chemrev.1c00755.

8. Brodszkij, E., Westensee, I.N., Holleufer, S.F., Ade, C., Andres, P.D.D., Pedersen, J.S., and Städler, B. (2022). Membrane composition of polymer-lipid hybrid vesicles. Appl. Mater. Today *29*, 101549. https://doi.org/10.1016/j.apmt.2022.101549.

9. Xu, Y., Yu, F., Jia, Y., Xu, X., and Li, J. (2024). Artificial Mitochondria Nanoarchitectonics via a Supramolecular Assembled Microreactor Covered by ATP Synthase. Angew. Chem. Int. Ed. Engl. *63*, e202411164. https://doi.org/10.1002/ANIE.202411164.

10. Noireaux, V., and Libchaber, A. (2004). A vesicle bioreactor as a step toward an artificial cell assembly. Proc. Natl. Acad. Sci. USA *101*, 17669–17674. https://doi.org/10.1073/pnas.0408236101.

11. Yue, K., Li, Y., Cao, M., Shen, L., Gu, J., and Kai, L. (2023). Bottom-up synthetic biology using cell-free protein synthesis. Adv. Biochem. Eng. Biotechnol. *185*, 1–20. https://doi.org/10.1007/10_2023_232.

12. Godino, E., and Danelon, C. (2023). Gene-Directed FtsZ Ring Assembly Generates Constricted Liposomes with Stable Membrane Necks. Adv. Biol. *7*, e2200172. https://doi.org/10.1002/adbi.202200172.

13. Ramirez-Diaz, D.A., Merino-Salomón, A., Meyer, F., Heymann, M., Rivas, G., Bramkamp, M., and Schwille, P. (2021). FtsZ induces membrane deformations via torsional stress upon GTP hydrolysis. Nat. Commun. *12*, 1–11. https://doi.org/10.1038/s41467-021-23387-3.

14. Osawa, M., and Erickson, H.P. (2013). Liposome division by a simple bacterial division machinery. Proc. Natl. Acad. Sci. USA *110*, 11000–11004. https://doi.org/10.1073/pnas.1222254110.

15. Zubaite, G., Hindley, J.W., Ces, O., and Elani, Y. (2022). Dynamic Reconfiguration of Subcompartment Architectures in Artificial Cells. ACS Nano *16*, 9389–9400. https://doi.org/10.1021/acsnano.2c02195.

16. Shetty, S.C., Yandrapalli, N., Pinkwart, K., Krafft, D., Vidakovic-Koch, T., Ivanov, I., and Robinson, T. (2021). Directed Signaling Cascades in Monodisperse Artificial Eukaryotic Cells. ACS Nano *15*, 15656–15666. https://doi.org/10.1021/acsnano.1c04219.

17. Yandrapalli, N., Petit, J., Bäumchen, O., and Robinson, T. (2021). Surfactant-free production of biomimetic giant unilamellar vesicles using PDMS-based microfluidics. Commun. Chem. *4*, 1–10. https://doi.org/10.1038/s42004-021-00530-1.

18. Moga, A., Yandrapalli, N., Dimova, R., and Robinson, T. (2019). Optimization of the Inverted Emulsion Method for High-Yield Production of Biomimetic Giant Unilamellar Vesicles. Chembiochem *20*, 2674–2682. https://doi.org/10.1002/cbic.201900529.

19. Ernits, M., Reinsalu, O., Yandrapalli, N., Kopanchuk, S., Moradpur-Tari, E., Sanka, I., Scheler, O., Rinken, A., Kurg, R., Kyritsakis, A., et al. (2024). Microfluidic production, stability and loading of synthetic giant unilamellar vesicles. Sci. Rep., 14071–1407114078. https://doi.org/10.1038/s41598-024-64613-4.

20. Van de Cauter, L., Jawale, Y.K., Tam, D., Baldauf, L., van Buren, L., Koenderink, G.H., Dogterom, M., and Ganzinger, K.A. (2024). High-Speed Imaging of Giant Unilamellar Vesicle Formation in cDICE. ACS Omega *9*, 42278–42288. https://doi.org/10.1021/acsomega.4c04825.

21. Sihorwala, A.Z., Lin, A.J., Stachowiak, J.C., and Belardi, B. (2023). Light-Activated Assembly of Connexon Nanopores in Synthetic Cells. J. Am. Chem. Soc. *145*, 3561–3568. https://doi.org/10.1021/jacs.2c12491.

22. de Matos, M.B.C., Miranda, B.S., Rizky Nuari, Y., Storm, G., Leneweit, G., Schiffelers, R.M., and Kok, R.J. (2019). Liposomes with asymmetric bilayers produced from inverse emulsions for nucleic acid delivery. J. Drug Target. *27*, 681–689. https://doi.org/10.1080/1061186X.2019.1579819.

23. Salibi, E., Peter, B., Schwille, P., and Mutschler, H. (2023). Periodic temperature changes drive the proliferation of self-replicating RNAs in vesicle populations. Nat. Commun. *14*, 1222–1229. https://doi.org/10.1038/s41467-023-36940-z.

24. Stephan, M.S., Dunsing, V., Pramanik, S., Chiantia, S., Barbirz, S., Robinson, T., and Dimova, R. (2023). Biomimetic asymmetric bacterial membranes incorporating lipopolysaccharides. Biophys. J. *122*, 2147–2161. https://doi.org/10.1016/j.bpj.2022.12.017.

25. Peter, B., Levrier, A., and Schwille, P. (2023). Spatiotemporal Propagation of a Minimal Catalytic RNA Network in GUV Protocells by Temperature

Cycling and Phase Separation. Angew. Chem. Int. Ed. Engl. *62*, e202218507. https://doi.org/10.1002/ANIE.202218507.

26. Sachet-Fernandez, G., Hindley, J.W., Ces, O., and Woscholski, R. (2024). Imidazole Headgroup Phospholipid Shows Asymmetric Distribution in Vesicles and Zinc-Dependent Esterase Activity. Biomolecules *14*, 1363. https://doi.org/10.3390/BIOM14111363/S1.
27. Fink, A., Doll, C.R., Yagüe Relimpio, A., Dreher, Y., Spatz, J.P., Göpfrich, K., and Cavalcanti-Adam, E.A. (2023). Extracellular Cues Govern Shape and Cytoskeletal Organization in Giant Unilamellar Lipid Vesicles. ACS Synth. Biol. *12*, 369–374. https://doi.org/10.1021/acssynbio.2c00516.
28. Adir, O., Albalak, M.R., Abel, R., Weiss, L.E., Chen, G., Gruber, A., Staufer, O., Kurman, Y., Kaminer, I., Shklover, J., et al. (2022). Synthetic cells with self-activating optogenetic proteins communicate with natural cells. Nat. Commun. *13*, 2328. https://doi.org/10.1038/s41467-022-29871-8.
29. Gonzales, D.T., Suraritdechachai, S., Zechner, C., and Tang, T.Y.D. (2023). Bidirectional Communication between Droplet Interface Bilayers Driven by Cell-Free Quorum Sensing Gene Circuits. ChemSystemsChem *5*, e202300029. https://doi.org/10.1002/syst.202300029.
30. Ji, Y., Chakraborty, T., and Wegner, S.V. (2023). Self-Regulated and Bidirectional Communication in Synthetic Cell Communities. ACS Nano *17*, 8992–9002. https://doi.org/10.1021/acsnano.2c09908.
31. Yang, C., Menge, J., Zhvania, N., Yu, M., Yang, H., Chen, D., Zheng, Z., Weitz, D.A., and Jahnke, K. (2025). Engineering Asymmetric Nanoscale Vesicles for mRNA and Protein Delivery to Cells. Adv. Funct. Mater. *35*, 2505738. https://doi.org/10.1002/adfm.202505738.
32. Yandrapalli, N., Seemann, T., and Robinson, T. (2020). On-Chip Inverted Emulsion Method for Fast Giant Vesicle Production, Handling, and Analysis. Micromachines *11*, 285. https://doi.org/10.3390/mi11030285.
33. Al Nahas, K., Fletcher, M., Hammond, K., Nehls, C., Cama, J., Ryadnov, M.G., and Keyser, U.F. (2022). Measuring Thousands of Single-Vesicle Leakage Events Reveals the Mode of Action of Antimicrobial Peptides. Anal. Chem. *94*, 9530–9539. https://doi.org/10.1021/acs.analchem.1c03564.
34. Rothschild, L.J., Averesch, N.J.H., Strychalski, E.A., Moser, F., Glass, J.I., Cruz Perez, R., Yekinni, I.O., Rothschild-Mancinelli, B., Roberts Kingman, G.A., Wu, F., et al. (2024). Building Synthetic Cells—From the Technology Infrastructure to Cellular Entities. ACS Synth. Biol. *13*, 974–997. https://doi.org/10.1021/acssynbio.3c00724.
35. Yandrapalli, N., and Robinson, T. (2019). Ultra-high capacity microfluidic trapping of giant vesicles for high-throughput membrane studies. Lab Chip *19*, 626–633. https://doi.org/10.1039/C8LC01275J.
36. Yandrapalli, N. (2025). Bottom-up development of lipid-based synthetic cells for practical applications. Trends Biotechnol. *43*, 2150–2169. https://doi.org/10.1016/j.tibtech.2025.03.009.
37. Cho, W., and Stahelin, R.V. (2005). Membrane-protein interactions in cell signaling and membrane trafficking. Annu. Rev. Biophys. Biomol. Struct. *34*, 119–151. https://doi.org/10.1146/annurev.biophys.33.110502.133337.
38. Jacob, V., Lambour, A., Swinyard, B., Zerbib, Y., Diouf, M., Soudet, S., Brochot, E., Six, I., Maizel, J., Slama, M., and Guillaume, N. (2023). Annexin-V positive extracellular vesicles level is increased in severe COVID-19 disease. Front. Med. *10*, 1186122. https://doi.org/10.3389/fmed.2023.1186122.
39. Shi, Z., Hu, M., and Bian, J. (2024). 99mTc-HYNIC-Annexin V detection of apoptosis in the rabbit model of liver cancer. Cell. Mol. Biol. *70*, 143–147. https://doi.org/10.14715/CMB/2024.70.7.20.
40. Hsu, Y.Y., Chen, S.J., Bernal-Chanchavac, J., Sharma, B., Moghimianavval, H., Stephanopoulos, N., and Liu, A.P. (2023). Calcium-triggered DNA-mediated membrane fusion in synthetic cells. Chem. Commun. *59*, 8806–8809. https://doi.org/10.1039/d3cc02204h.
41. Dziubak, D., and Sęk, S. (2023). Sparsely tethered bilayer lipid membranes formed by self-assembly of bicelles: Spectroelectrochemical characterization and incorporation of transmembrane protein. Bioelectrochemistry *153*, 108482. https://doi.org/10.1016/J.BIOELECHEM.2023.108482.
42. Van de Cauter, L., Fanalista, F., van Buren, L., De Franceschi, N., Godino, E., Bouw, S., Danelon, C., Dekker, C., Koenderink, G.H., and Ganzinger, K.A. (2021). Optimized cDICE for Efficient Reconstitution of Biological Systems in Giant Unilamellar Vesicles. ACS Synth. Biol. *10*, 1690–1702. https://doi.org/10.1021/acssynbio.1c00068.
43. Chiba, M., Miyazaki, M., and Ishiwata, S. (2014). Quantitative Analysis of the Lamellarity of Giant Liposomes Prepared by the Inverted Emulsion Method. Biophys. J. *107*, 346–354. https://doi.org/10.1016/J.BPJ.2014.05.039.
44. Schönnenbeck, P., Junglas, B., and Sachse, C. (2025). CryoVIA: An image analysis toolkit for the quantification of membrane structures from cryo-EM micrographs. Structure *33*, 808–819.e4. https://doi.org/10.1016/j.str.2025.01.013.
45. Alsayyah, C., Rodrigues, E., Hach, J., Renne, M.F., and Ernst, R. (2025). Reversible tuning of membrane sterol levels by cyclodextrin in a dialysis setting. Biophys. J. *124*, 1433–1445. https://doi.org/10.1016/j.bpj.2025.03.020.
46. Bligh, E.G., and Dyer, W.J. (1959). A RAPID METHOD OF TOTAL LIPID EXTRACTION AND PURIFICATION. Can. J. Biochem. Physiol. *37*, 911–917. https://doi.org/10.1139/o59-099.

STAR★METHODS

KEY RESOURCES TABLE

REAGENT or RESOURCE	SOURCE	IDENTIFIER
Bacterial and virus strains		
E. coli DH5α	NEB	C2987
E. coli BL21 (DE3)	NEB (C2527)	C2527
Chemicals, peptides, and recombinant proteins		
L-Alanine	Sigma, USA	A7627
L-Arginine	Sigma, USA	A5006
L-Asparagine	Sigma, USA	A0884
L-Aspartic acid	Sigma, USA	A9256
L-Cysteine	Sigma, USA	W326305
L-Glutamic acid	Sigma, USA	G1251
L-Glutamine	Sigma, USA	G3126
Glycine	Sigma, USA	G7126
L-Histidine	Sigma, USA	H8000
L-Isoleucine	Sigma, USA	I2752
L-Leucine	Sigma, USA	L8000
L-Lysine	Sigma, USA	L5501
L-Methionine	Sigma, USA	M9625
L-Phenylalanine	Sigma, USA	P2126
L-Proline	Sigma, USA	P0380
L-Serine	Sigma, USA	S4500
L-Threonine	Sigma, USA	T8625
L-Tryptophan	Sigma, USA	T0254
L-Tyrosine	Sigma, USA	T3754
L-Valine	Sigma, USA	V0500
Sucrose	Sigma, USA	1076539053
Glucose	Sigma, USA	G8270
NTPs		N/A
Adenosine 5′-triphosphate disodium salt hydrate	Sigma, USA	A26209
Cytidine 5′-triphosphate disodium salt hydrate	Sigma, USA	30320
Guanosine 5′-triphosphate sodium salt hydrate	Roche, CH	10106399001
Uridine 5′-triphosphate trisodium salt dihydrate	Sigma, USA	94370
β- Nicotinamide adenine dinucleotide (NAD)	Sigma, USA	N1511
Coenzyme A (CoA)	Sigma, USA	C4284
Folinic acid calcium salt hydrate	Sigma, USA	F7878
Oxalic acid	Roth, DE	8879.1
Phosphoenolpyruvate (PEP)	Roche, CH	10108294001
Putrescine	Sigma, USA	51799
Spermidine	Sigma, USA	Sigma, USA
tRNA from *E. coli* MRE600	Roche, CH	10109541001
Acetic acid (HOAc)	Merck, USA	K48001663 632
Dithiothreitol (DTT)	Thermo, USA	R0862
HEPES (N-2-Hydroxyethylpiperazine-N′-2- ethane sulphonic acid)	Carl Roth, DE	9105
L-Glutamic acid hemimagnesium salt tetrahydrate	Sigma, USA	49605
L-Glutamic acid potassium salt monohydrate	Sigma, USA	G1149

(*Continued on next page*)

Continued

REAGENT or RESOURCE	SOURCE	IDENTIFIER
Potassium hydroxide	Sigma, USA	221473
Potassium phosphate dibasic solution	Sigma, USA	P8584
Potassium phosphate monobasic solution	Sigma, USA	P8709
Trizma base	Sigma, USA	T1503
Mineral Oil	Sigma, USA	M5904
1,2-dioleoyl-*sn*-glycero-3-phosphocholine (DOPC)	Avanti, USA	850356C
Biotin PE	Avanti, USA	860562C
1,2-diheptanoyl-*sn*-glycero-3-phosphocholine (DHPC)	Avanti, USA	850306
Cholesterol (Chol)	Sigma, USA	C3045
N-(3-oxohexanoyl)-L-homoserine lactone (AHL)	Sigma, USA	K3007
1,1′-dioctadecyl-3,3,3′,3′-tetramethylindodicarbocyanine, 4-chlorobenzenesulfonate salt (DiD)	ThermoFisher, USA	D7757
Calcein	Sigma, USA	C0875
Streptavidin, Alexa Fluor™ 488 Conjugate	ThermoFisher, USA	S32354
Annexin V CF®488A	Biotium	29005
PETG TRANSPARENT	Azurefilm	AFPETGT
Glass coverslips, 24 × 60 mm, Thickness No. 1.5H	Marienfeld SUPERIOR	01 072 42
R&G EPO5. F250A + B 5-Minute Epoxy Resin	Faserverbundwerkstoffe Composite Technology	EPO5. F250A + B
**Critical commercial assays**		
NEBuilder HiFi DNA Assembly Kit	NEB, USA	E2621
QIAprep Spin Miniprep Kit	QIAGEN, DE	27104
QIAGEN Plasmid Maxi Kit	QIAGEN, DE	12162
QIAquick PCR Purification Kit	QIAGEN, DE	28104
myTXTL Sigma 70 Master Mix Kit	Daicel Arbor Biosciences, USA	507024
**Deposited data**		
Confocal and Electron microscopy data	Mendeley data	https://doi.org/10.17632/rx7bkm4mtb.1
**Experimental models: Cell lines**		
HEK293T cells	ATCC	CRL-3216
**Recombinant DNA**		
pEXP5-NT/pT7 LuxR	Gonzales et al. (2023)[29]	N/A
pEXP5-NT/pLux EGFP	Gonzales et al. (2023)[29]	N/A

METHOD DETAILS

Plasmids constructed in this study used the pEXP5-NT vector backbone of the pEXP5-NT/6xHis eGFP plasmid. Vector and insert parts were made by PCR amplification and overlap extension (NEB Phusion HF). Plasmid assembly of the vector and insert parts were undertaken using Gibson assembly using the NEBuilder HiFi DNA Assembly Kit (NEB, USA) or restriction digest and ligation. All plasmids were prepared and purified by ethanol precipitation from E. Coli DH5α cultures using the QIAGEN Plasmid Maxi Kit (QIAGEN, Germany) and measured by NanoDrop 2000 (Thermo, USA). All PCR, purification, and assembly methods were performed using the manufacturer's standard protocols. All primers, ultramers, and gBlocks were synthesized by Integrated DNA Technologies (IDT, www.idtdna.com). Plasmid assembly was confirmed by Sanger sequencing (GENEWIZ, www.genewiz.com) and deposited in Addgene (addgene.org) with plasmid Ids 193624–193631.

E. coli extract

The following solutions were prepared.

- LB Media: 200 mL

- 2xYTP Media: 1 L of a solution containing 5 g NaCl, 10 g yeast extract, 16 g tryptone, 40 mL of 1 M potassium phosphate dibasic solution, and 22 mL of 1 M potassium phosphate monobasic solution. The final volume was adjusted to 1 L with water.
- S30A Buffer: 500 mL of a buffer with 14 mM MgGlu, 60 mM KGlu, 50 mM Tris, and 2 mM DTT, adjusted to pH 7.7 using approximately 1 mL of glacial acetic acid.
- S30B Buffer: 1 L of a buffer containing 14 mM MgGlu, 60 mM KGlu, and 2 mM DTT, adjusted to pH 8.2 with about 2 mL of 2 M Tris.
- DTT: 1 mL of 1 M DTT.

E. coli cell-free extract preparation

All steps were performed on ice or at 4°C unless noted otherwise.

1. Starter Culture: An overnight culture of *E. coli* BL21 (DE3) cells was grown in 200 mL of LB media at 37°C with 180 rpm shaking.
2. Production Culture: The starter culture was used to inoculate two separate 500 mL pre-warmed 2xYTP media cultures to a starting optical density (OD600) of 0.05. The cultures were incubated at 37°C with 180 rpm shaking for approximately 3–5 h until they reached an OD600 of 1.6.
3. Cell Harvesting: The cells were harvested by centrifuging the cultures at 5000 xg for 10 min.
4. Washing: The resulting cell pellet was scooped into a pre-weighed 50 mL tube and washed three times with 30 mL of S30A buffer.
5. Pellet Storage: The washed cell pellet was weighed (a typical yield is around 2.5g of wet pellet per 500 mL of culture), flash-frozen in liquid nitrogen, and stored overnight at −80°C.
6. Resuspension: The next day, the frozen pellet was thawed and resuspended by vortexing in S30A buffer at a ratio of 1 mL of buffer per 1 g of wet pellet.
7. Aliquot Preparation: The resuspended cell solution was divided into 1.5 mL aliquots in 2 mL microcentrifuge tubes.
8. Cell Lysis (Sonication): The cells were lysed via sonication in an ice-water bath to prevent overheating. This was done in 10 cycles, with each cycle consisting of a 10-s pulse and a 30-s rest at 25% amplitude. The resulting lysate should appear darker brown.
9. DTT Addition: Immediately after sonication, 2 μL of 1M DTT was added to each 2 mL microcentrifuge tube.
10. Initial Clarification: The sonicated lysates were centrifuged at 18,000 xg for 10 min.
11. Run-off Reaction: The clear supernatant was collected, pooled into a 10 mL tube, and incubated with the cap open at 37°C and 250 rpm for 1 h. This step allows for the degradation of unwanted mRNA transcripts and proteins that could interfere with subsequent reactions.
12. Second Clarification: The lysate was clarified by centrifuging it again at 10,000 xg for 10 min to remove any remaining precipitates.
13. Dialysis: The clarified lysate was dialyzed against S30B buffer for 3 h at 4°C using a dialysis membrane with a 12–14 kDa molecular weight cut-off.
14. Final Clarification: The dialyzed lysate was centrifuged at 10,000 xg for 10 min.
15. Storage: The final lysate was aliquoted into 50 μL volumes, flash-frozen in liquid nitrogen, and stored at −80°C for future use.

Solution A

The stock and final concentrations of the components used for Solution A are listed in Table 2. All stock solutions were prepared by dissolving in water and the HEPES buffer was adjusted to pH 7 using KOH. 50 μL aliquots of Solution A were flash frozen with liquid nitrogen and stored at −80°C until use.

Solution B

The stock and final concentrations of the components of Solution B are listed in Table 2. The amino acid mix contains 50 mM of each amino acid and was incubated at 37°C with shaking to help dissolve the powder. Note that the amino acids may not dissolve completely in solution. The amino acid mix is prepared in a large volume of 10 mL to ensure that sufficient material could be weighed out. The amino acid solution should be fully mixed before adding into the final Solution B mixture. The stock of magnesium glutamate and potassium glutamate were prepared as one solution. The PEP stock solution was adjusted to pH 7 using 10 M KOH solution. The amounts of the final Solution B mix are given to make 2.1 mL of Solution B or approximately 1000 reactions. 50 μL aliquots of Solution B were flash frozen with liquid nitrogen and stored at −80°C until use.

The CFES reaction mix

The final *E. coli* extract-based CFES master mix is prepared according to Table 3 and incubated at 25°C–37°C for 5–10 h. The amount of magnesium glutamate supplemented to the final CFES optimized to be 2mM. To prepare the CFES mix, the DNA + water components of each sample were first pre-mixed, the remainder of the master mix was then added to avoid long waiting times between starting the CFE reactions.

Preparation of lipid extracts from mammalian cell culture

HEK293T cells (available at ACTT, product No.: CRL-3216) were cultivated in 15 cm dishes to ~80% confluency in DMEM high glucose medium (GIBCO, product No.: 41966052) supplemented with 10% FBS (PAN-Biotech, product No.: P30-3033).

Isolation of whole cell lipids from HEK293T cells

After the removal of the cell culture medium, HEK293T cells from five 15 cm cell culture dishes were resuspended in 5 mL PBS. The resulting cell suspension was centrifuged (700 x g, 5 min, room temperature), the supernatant was discarded, and the cells in the pellet were subjected to lipid extraction.

Isolation of lipids from mitochondria-enriched, crude microsomes

Mitochondria-enriched, crude microsomes were isolated as previously described.[45] Briefly, HEK293T cells were cultivated in five 15-cm cell culture dishes to ~80% confluency. Cells were harvested in 5 mL of cold PBS per plate. Cells were pelleted by centrifugation (500 x g, 5 min, 4°C) before adding the equivalent of 10 cell volumes of hypotonic buffer (20 mM HEPES pH 7.5, 5 mM KCl, 1.5 mM MgCl2, 2 mM DTT, 0.03 mg/mL protease inhibitor cocktail). The cells were incubated on ice for 15 min and occasionally agitated. Cells were then lysed on ice by 35 strokes in a pre-cooled glass Dounce homogenizer with a tight-fitting pestle. The lysate was mixed with 2.5 volumes of membrane buffer 1 (20 mM HEPES pH 7.5, 525 mM mannitol, 175 mM sucrose, 5 mM EDTA, 2 mM DTT, 0.03 mg/mL protease inhibitor cocktail) by gentle inversion. Intact cells, nuclei, and cell debris were removed by two consecutive centrifugations (700 x g, 10 min, 4°C). The final supernatant fraction was also centrifuged twice (10,000 x g, 10 min, 4°C) to yield a crude microsome fraction enriched for mitochondrial membranes in the pellet. Microsomes were resuspended in ~200 μL of membrane buffer 2 (20 mM HEPES pH 7.5, 210 mM mannitol, 70 mM sucrose, 0.5 mM EDTA, 2 mM DTT, 0.03 mg/mL protease inhibitor cocktail) and aliquoted into portions of 50 μL prior to lipid extraction.

Total lipid extraction

Lipids were extracted using a modified two-step Bligh and Dyer extraction.[46] Briefly, each 50 μL sample was mixed with 150 μL of ammonium bicarbonate solution (150 mM). After the addition of 750 μL of chloroform:methanol (2:1), the sample was rigorously agitated for 15 min at 25°C,1400 rpm. A subsequent addition of 250 μL of chloroform and 250 μL of ammonium bicarbonate (150 mM) induced phase separation. The sample was agitated for 15 min at 25°C at 1400 rpm. The organic phase was collected after a brief centrifugation (2000 x g, 2 min, room temperature). The aqueous phase was subjected to a second round of lipid extraction after adding 500 μL chloroform. The organic phases from both rounds of extraction were pooled in a glass tube, and brought into a nitrogen atmosphere before sealing the tube and storage at −20°C.

Thin-layer chromatography (TLC)

Lipid separation on a thin-layer chromatography (TLC) Silica 60 gel layer ADAMANT (Macherey-Nagel) was performed using chloroform:methanol:H2O at a 70:25:2 ratio (v/v) as the mobile phase. Exactly 1 μL of the following lipids were used as standards: PC (25 mg/mL), PE (25 mg/mL) and PS (25 mg/mL). 25 μL of crude microsomes (A280 of 22) were subjected to lipid extraction and total lipids were spotted on the plate. After migration, the silica plates were dried in a chemical hood for 15 min and then stained for 10–15 min with iodine. The silica plates were scanned on an EPSON V750 PRO (see Figure S7).

Inverted emulsion method

A clear step-by-step video for reproducible production vesicles using this method is attached as Video S6.

(i) Lipid-oil preparation: Lipid-oil dispersions were prepared using mineral oil as the base solvent. Lipids at a desired concentration of 600 μM, initially dispersed in chloroform, were dried in a glass vial under a stream of nitrogen and subsequently placed in a desiccator under low pressure for 45 min to ensure complete chloroform removal. After drying, 2 mL of mineral oil was added to the vial, and the mixture was subjected to bathe sonication for 15 min at room temperature. The resulting lipid-oil dispersion was then stored at 4°C until use.
(ii) Coating the Chambered slides: Chambered slides were coated using β-casein (2 mg/mL) to prepare the ibidi chambered slides (μ-Slide VI 0.5 Glass Bottom, Cat no. 80607) with a glass coverslip. A volume of 100 μL of β-casein solution was gently pipetted into each chamber. If bubbles formed, the slides were centrifuged at 900 ×g for 3 min to ensure uniform coating. The slides were then incubated for 2 h or until the solution had dried in a desiccator under low-pressure conditions.
(iii) Formation of interfacial lipid-monolayer: To form an interfacial lipid monolayer, the β-casein-coated slide was rinsed twice with 100 μL of the outer solution, which serves as the dispersing medium for the synthetic cells. After rinsing, 100 μL of the outer solution was added to the slide. If air bubbles formed, the slide was centrifuged at 900 ×g for 3 min or 2000 ×g for 5 min to ensure uniform coverage. Following this, 25 μL of the 600 μM lipid-oil solution was added to one chamber, while 25 μL of the outer solution was added to the other chamber. The slide was then incubated for 30 min to allow for the formation of the interfacial lipid monolayer.
(iv) Preparation of Water-in-Oil emulsion: To prepare a water-in-oil (W/O) emulsion, 200 μL of a 600 μM lipid-oil solution was pipetted into a 1.5 mL Eppendorf tube. Subsequently, 5 μL of the inner solution, which forms the inner volume of the synthetic

cells, was added to the tube. The tube was then ratchetted using an Eppendorf rack to generate water-in-oil droplets. For the preparation of sub-micrometer W/O emulsions suitable for electron microscopy (EM) imaging, a magnetic stirring method was employed. In this approach, a small magnetic bead was added to a glass vial containing 200 μL of the 600 μM lipid-oil solution and 5 μL of the inner solution, and the mixture was stirred at 2000 rpm for 10 min instead of using the ratchetting method.

(v) Centrifugation: For the centrifugation step, 25 μL of the prepared water-in-oil (W/O) emulsion was immediately pipetted into the chamber where the interfacial lipid monolayer had formed. Simultaneously, 25 μL of the outer solution was added to the adjacent chamber. Centrifugation was then performed at varying speeds, depending on the density of the inner and outer solutions used, to facilitate the formation of synthetic cells.

Thin film hydration with sonication

Lipid-coated glass vial is prepared by adding POPC lipids in chloroform and blow dried with nitrogen gas followed by desiccation for 45 min at negative pressure. This thin film is hydrated using PBS at 37°C for 2 h before probe sonication. To achieve nanometre sized liposomes, the entire sample was probe sonicated at 30 W for 20 min with 10 s intervals for every 20 s, in the presence of ice.

Confocal microscopy

Vesicles are imaged using Leica SP8 fitted with HC PL APO 63x/1,30 GLYC CORR CS2 objective. For data acquisition, 488 nm excitation wavelength (eGFP, Calcein, Streptavidin Alexa Fluor 488) is used with filters at 520 ± 20 nm emission wavelengths. In the case of DiD dye labeling the membrane, 637 nm excitation wavelength is used with filters at 650 ± 20 nm emission wavelengths. All time-lapses are acquired with 512 × 512 resolution every 5 min.

Electron microscopy

4 mL of dispersion were rapidly plunge-frozen on Quantifil Holey Carbon grids (R 3.5/1) using a Leica Automatic Plunge Freezer EM GP. Samples were imaged with a FEI Titan Halo (Thermo Fisher Scientific) operated at 300 keV using a Gatan energy filter and a Gatan K2 Summit direct electron detector. Serial EM software was used to operate the microscope during acquisition. The acquired micrographs are analyzed using CryoVia tool-kit.[44] A detailed methodology can be found here.[44] The bilayer thickness is plotted using OriginPro 2025 (OriginLabs).

3D printable chip production

The CAD designs are produced using Tinker CAD website and imported into the Bamboo studio software from Bamboo Lab connected to Bamboo X1-Carbon 3D printer. The infill density is set to 100% for robustness and 0.12 mm nozzle is used to 3D print the chip using standard PETG filament. Channel designs are supplied as a supplementary data file. Upon printing, the chip is bonded to the 0.17 mm glass coverslip of same dimension. For bonding, a pea-sized epoxy (R&G EPO5. F250A + B 5-Minute Epoxy Resin, Faserverbundwerkstoffe composite technology, Germany) is mixed freshly and smeared to the plastic chip. After evenly spreading the epoxy, the plastic chip is bonded to the glass coverslip. The entire assembly is allowed to settle for 24 h before using that provides customizability without compromising the simplicity of the method. Vesicles were prepared in these channel devices and analyzed as previously described. The time taken for the diffusion of calcein, after injection, was determined by confocal microscopy with time resolved detection of calcein λ_{exc}: 488 nm and λ_{em}:520 ± 20 nm and plot as a function of the channel length.

Analysis of vesicle loss and contamination due to extraction through the oil phase

To compare the relative loss of vesicles and contamination from extraction lipid vesicles were prepared by the thin-film hydration method with a lipid composition of 99% POPC and 1% NBD-PE. A lipid-coated glass vial is prepared by adding 1 mM total lipid concentration in chloroform followed by drying with a steady stream nitrogen gas and desiccation for 45 min at negative pressure. This thin film is hydrated using 600 mOsm sucrose solution at 37°C for 2 h and vortexed before using. 5 μL of this suspension was loaded into a chip, the well plate and an Eppendorf with 100 μL glucose solution and the vesicles were allowed to settle. 50 μL of mineral oil containing 5% DiD was loaded over the aqueous dispersion. A reference sample was prepared with vesicles in glucose solution in an Eppendorf tube without the oil layer on top. The vesicles were extracted from the aqueous dispersion either through the oil layer for the well plate and Eppendorf or through the second well from the chip and loaded into into a Attune NxT Flow cytometer (Thermo Fischer). The vesicles were detected by fluorescence of NBD λ_{exc}: 488 nm and λ_{em}: 520 ± 20 nm as well as DiD λ_{exc} at 637 nm and λ_{em} 670 ± 20 nm. The retrieval % and % contamination was determined from 3 repeat experiments. Simultaneous detection of NBD and DiD provided the percentage of vesicle population that was contaminated with mineral oil as a consequence of retrieval.

QUANTIFICATION AND STATISTICAL ANALYSIS

The vesicles were randomly chosen; luminal fluorescence is reported without area/volume normalization. Statistical analyses were performed using OriginLabs OriginPro 16 software. For fitting the decay and expression curves in Figure 3. Time-dependent

fluorescence data were analyzed in OriginPro (OriginLab). The decay of the intravesicular fluorescence after addition of α-hemolysin (Figure 3F) was fitted with a exponential decay function (Origin "ExpDec1"):

$$I(t) = I_\infty + (I_0 - I_\infty)\exp(-t/\tau),$$

where I_0 is the initial intensity, I_∞ is the residual intensity at long times, and τ is the characteristic time constant. This expression is the solution of a first-order rate equation $dI/dt = -k(I-I_\infty)$, which describes pore-mediated leakage where the efflux rate is proportional to the amount of fluorescent dye remaining inside the vesicle ($\tau = 1/k$).

The increase of eGFP fluorescence inside vesicles (Figure 3H) was fitted with a Boltzmann sigmoidal function (Origin "Boltzmann"):

$$I(t) = I_{\min} + \frac{I_{\max} - I_{\min}}{1+\exp\left(\frac{t_0 - t}{\Delta t}\right)},$$

where I_{min} and I_{max} are the lower and upper asymptotes, t_0 is the half-time of the increase, and Δt characterizes the steepness of the transition. This phenomenological model captures the expected behavior of cell-free protein expression in vesicles, with an initial lag phase, a rapid production phase, and a final saturation when resources become limiting. All results are shown as mean ± SD.

Cell Reports Methods, Volume *6*

Supplemental information

A robust method for on-chip production and manipulation of lipid vesicles by inverted emulsion

Naresh Yandrapalli, David T. Gonzales, Weihua Leng, Cynthia Alsayyah, Nurzhan Abdukarimov, Robert Ernst, and T.-Y. Dora Tang

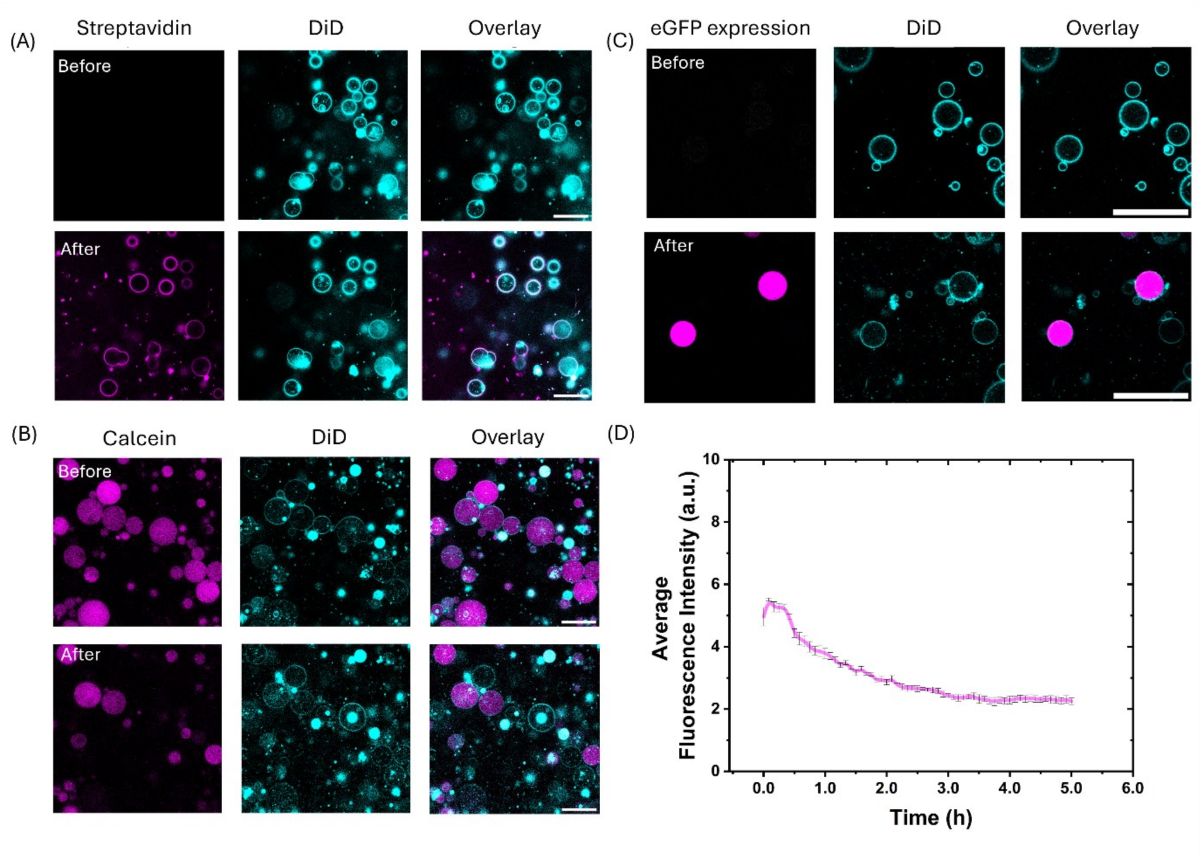

Figure S1: Diffusion-assisted manipulation of synthetic cells. (A) Confocal microscopy images of Streptavidin binding to vesicles with PE-biotin, before and after 30 min of incubation with 50 nM labelled Streptavidin protein. Scale bar – 50 µm. (B) Confocal snapshots of calcein containing vesicles before and after 3 h of incubation with 1µg/mL alpha-hemolysin membrane pore protein. Scale bar – 50 µm. (C) Confocal snapshots of b-CFES containing vesicles before and after 6 h of incubation with 1µM acyl-homoserine lactone. Scale bar – 50 µm. (D) Average luminal fluorescence intensity observed for vesicles with b-CFES that are uninduced. The timelapse plot suggests no increase in fluorescence intensity up to 5 h. Data is shown as mean ± SD. Related to Figure 3.

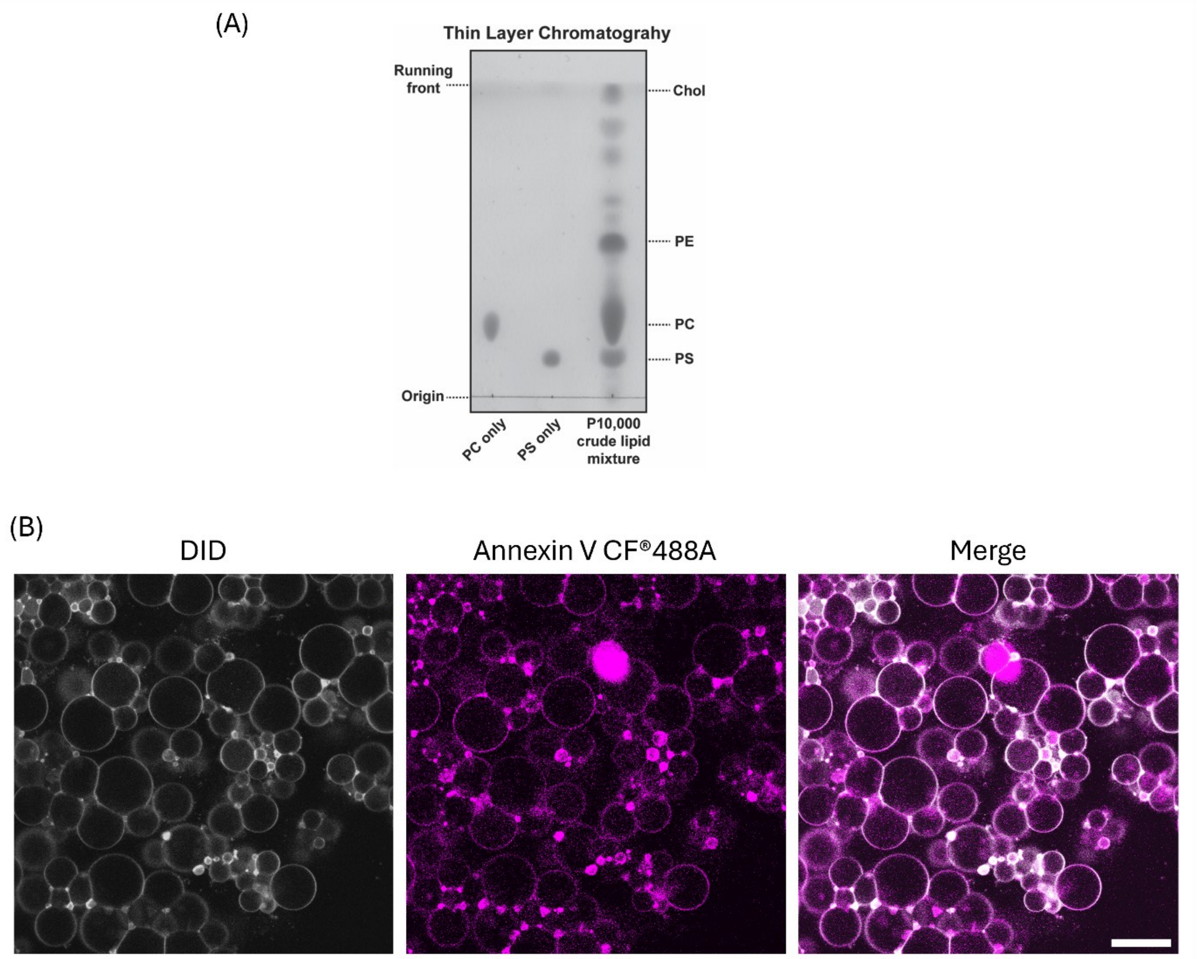

Figure S2: Synthetic cells production using mammalian lipid extracts. (A) Validation of crude lipid mixtures derived from HEK293T cells. Crude lipid extracts from mitochondria-enriched microsomes (P10.000) were subjected to TLC together with POPC (1 µL, 25 mg/mL) and POPS (1 µL, 25 mg/mL) and POPE (1 µL, 25 mg/mL) standards. Lipids were separated using chloroform:methanol:H_2O (70:25:2) as the mobile phase and stained with iodine. (B) Confocal image of bound Annexin V to PS containing synthetic cells with a lipid composition comprising of membrane pellet lipids extracted from mammalian HEK293T cells. Scale bar – 20 µm. Related to Figure 3

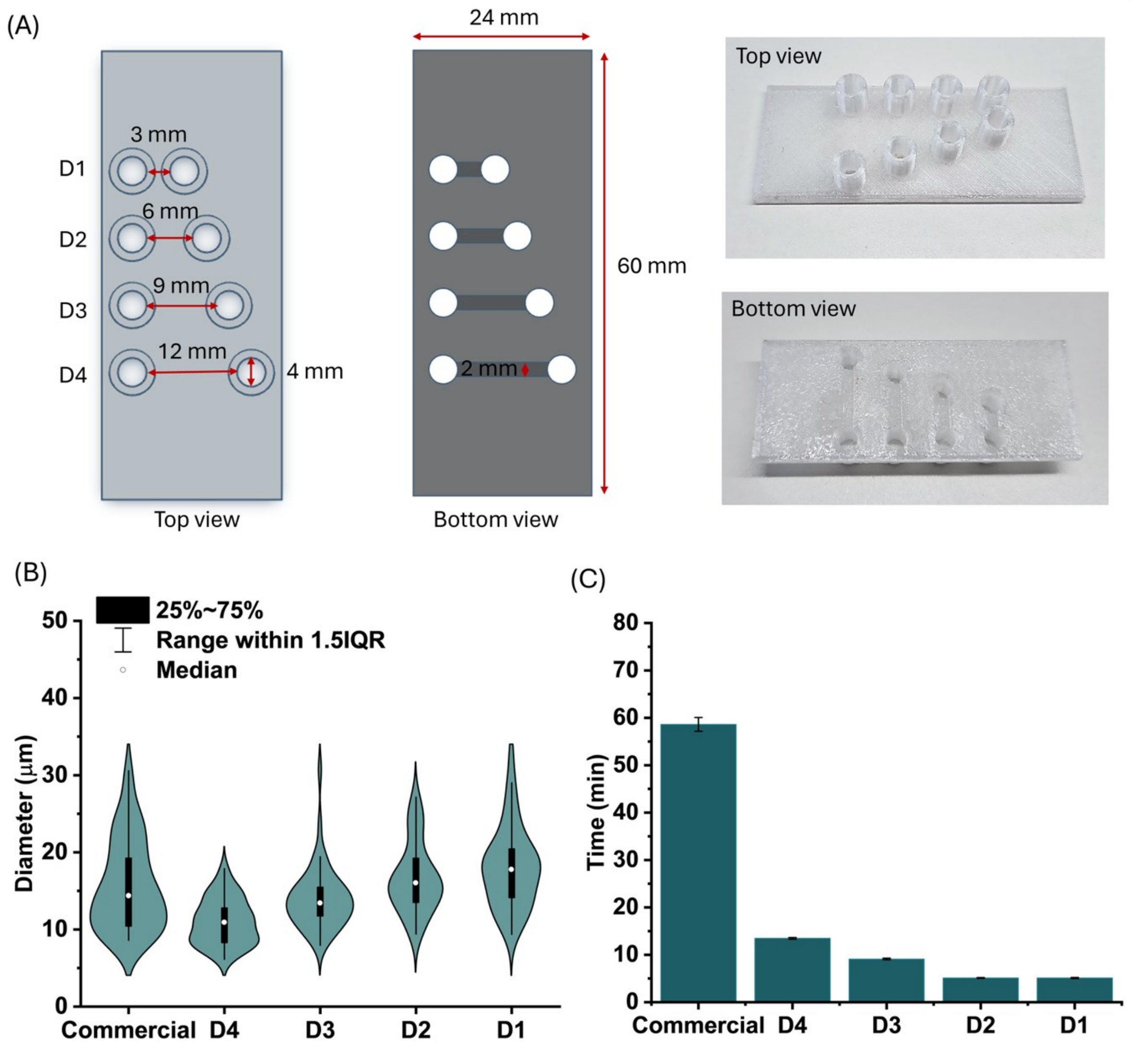

Figure S3: 3D printed chip design. (A) Schematics of the 3D printable chip design with varying channel lengths, D1 being the shortest and D4 the longest (left). Snapshots of 3D printed chips with top view and bottom view (left) after assembly with glass coverslip. (B) Comparative size analysis of size distribution of vesicles produced from commercial ibide® chips and 3D printed chip with four different distances are presented as violine plots obtained from n≥35. With a mean diameter around within 10-20 μm, has coefficient of variation, from left to right, 44%, 33%, 22%, 23% and 27%. (C) Diffusion time can be modulated depending on the length of the channel. The shorter the channel the faster the diffusion. Related Figure 1 & 2.

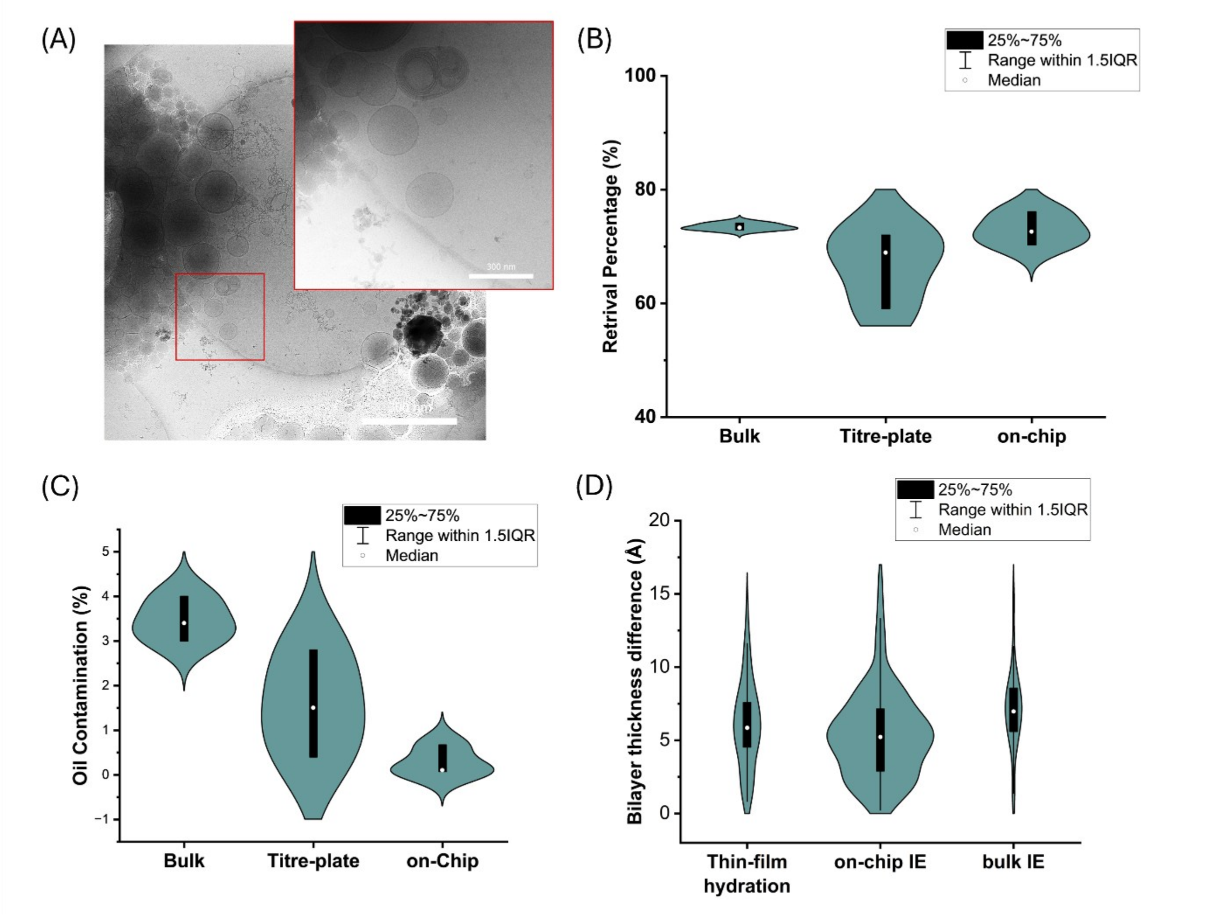

Figure S4: Systematic evaluation of synthetic cells after retrieval. (A) Wide field of view (11500x) electron micrograph of lipid vesicles, inset showing the close-up (48000x) of lipid vesicles. Scale bar corresponds to 300 nm. (B) Comparative flow cytometry analysis of number of vesicles retrieved from Eppendorf tubes vs Microtitre 96-well plate vs on-chip methods. The average retrieval percentage corresponds to 73.4±0.6%, 66±6%, and 73±2.9% for all the methods, respectively. In each of these cases vesicles were removed through an oil layer. Our results show that vesicles are lost during retrieval through oil regardless of the vessel they are contained. **(C).** DiD-based percentage oil contamination of vesicles retrieved using three different methods was evaluated using Flow cytometry. The average contamination from retrieval via an oil layer from an Eppendorf®-based method (bulk) or microtiter 96-well plate (titre plate) showed 8.8±0.98% and 4.1±2.5% contamination respectively. Retrieval from the second well of the chip (on chip) without an oil layer showed 0.97 ± 1.2% contamination. The violine plots are obtained using three replicates for all three methodologies. (D) Distribution of the bilayer thickness difference amongst vesicles produced using different methods. The on-chip IE and thin-film hydration methods produce membranes with a lower median asymmetry (~5-6 Å) compared to the bulk IE method (~7 Å) (n≥50). Related to Figure 4.
